# Robust Transcriptional Response to Heat Shock Impacting Diverse Cellular Processes despite Lack of Heat Shock Factor in Microsporidia

**DOI:** 10.1128/mSphere.00219-19

**Published:** 2019-05-22

**Authors:** Nora K. McNamara-Bordewick, Mia McKinstry, Jonathan W. Snow

**Affiliations:** a Biology Department, Barnard College, New York, New York, USA; Carnegie Mellon University

**Keywords:** heat shock factor, honey bee, *Nosema*, heat shock response, microsporidia, proteostasis

## Abstract

The majority of fungal species prefer the 12° to 30°C range, and relatively few species tolerate temperatures higher than 35°C. Our understanding of the mechanisms underpinning the ability of some species to grow at higher temperatures is incomplete. Nosema ceranae is an obligate intracellular fungal parasite that infects honey bees and can cause individual mortality and contribute to colony collapse. Despite a reduced genome, this species is strikingly thermotolerant, growing optimally at the colony temperature of 35°C. In characterizing the heat shock response (HSR) in N. ceranae, we found that this and other microsporidian species have lost the transcriptional regulator HSF and possess a reduced set of putative core HSF1-dependent HSR target genes. Despite these losses, *N. ceranae* demonstrates robust upregulation of the remaining HSR target genes after heat shock. In addition, thermal stress leads to alterations in genes involved in various metabolic pathways, ribosome biogenesis and translation, and DNA repair. These results provide important insight into the stress responses of microsporidia. Such a new understanding will allow new comparisons with other pathogenic fungi and potentially enable the discovery of novel treatment strategies for microsporidian infections affecting food production and human health.

**IMPORTANCE** We do not fully understand why some fungal species are able to grow at temperatures approaching mammalian body temperature. Nosema ceranae, a microsporidium, is a type of fungal parasite that infects honey bees and grows optimally at the colony temperature of 35°C despite possessing cellular machinery for responding to heat stress that is notably simpler than that of other fungi. We find that N. ceranae demonstrates a robust and broad response to heat shock. These results provide important insight into the stress responses of this type of fungus, allow new comparisons with other pathogenic fungi, and potentially enable the discovery of novel treatment strategies for this type of fungus.

## Abstract (precis)

We do not fully understand why some fungal species are able to grow at temperatures approaching mammalian body temperature. Nosema ceranae, a microsporidium, is a type of fungal parasite that infects honey bees and grows optimally at the colony temperature of 35°C despite possessing cellular machinery for responding to heat stress that is notably simpler than that of other fungi. We find that N. ceranae demonstrates a robust and broad response to heat shock. These results provide important insight into the stress responses of this type of fungus, allow new comparisons with other pathogenic fungi, and potentially enable the discovery of novel treatment strategies for this type of fungus.

## INTRODUCTION

Fungal species are highly varied in their tolerance to high temperature, although the majority prefer the range of 12° to 30°C, and relatively few species tolerate temperatures higher than 35°C (1). This bottleneck of species able to grow at higher temperatures is thought to limit the number of fungal species able to colonize animals with higher body temperatures, such as mammals. Although thermal stress is known to have broad effects on the cellular processes of other fungal species, including Saccharomyces cerevisiae (reviewed in reference 2), Candida albicans (3), Schizosaccharomyces pombe (4), Aspergillus fumigatus (5), and Metarhizium anisopliae (6), our understanding of the mechanisms underpinning the ability of some species to grow at higher temperatures is incomplete (7).

Microsporidia constitute a group of spore-forming unicellular obligate intracellular parasites which have recently been reclassified as fungi. Currently, approximately 1,500 species are known. Microsporidian infections are widespread in nature but are relatively understudied compared to other fungal groups (reviewed in reference 8). The microsporidian species *N. ceranae* has received considerable attention recently (10, 11) in response to intensifying the focus on the role of microbial attack on honey bee health (12). Comparative genomics indicates that *N. ceranae,* and microsporidia more broadly, have lost many of the cellular processes and pathways found in free-living eukaryotes (13). Yet, despite this genome compaction, *N. ceranae* exhibits a striking ability to grow at the high temperatures (34 to 35°C) maintained in honey bee colonies (14, 15).

We hypothesized that the genomic reduction in conjunction with selection for thermotolerance in this species could result in novel structure and function of the heat shock response (HSR), which responds to proteostatic disruption in the cytoplasm (16, 17). Our genomic analysis revealed that while some of the core components of the pathway are conserved, this species possesses reduced numbers of proteotoxic stress-related genes in comparison with other fungal species. Interestingly, we found that *N. ceranae* and other microsporidian species have lost the transcriptional regulator HSF that is critical for HSR function in other species. However, using RNA sequencing (RNA-seq), we found that *N. ceranae* possesses a robust induction of the remaining HSR target genes after heat shock. In addition, thermal stress leads to alterations in genes involved in various metabolic pathways, ribosome biogenesis and translation, and DNA repair. Finally, heat shock induces a significant number of genes encoding proteins of unknown function. These results provide an important new understanding of microsporidian cell biology and shed new light on how stress responses in these species compare to other pathogenic fungi. Moreover, the application of such discoveries to the treatment of microsporidian infections could have important impacts on food production and human health.

## RESULTS

### Heat shock can dramatically reduce *Nosema* infection intensity in honey bees.

**FIG 1:**
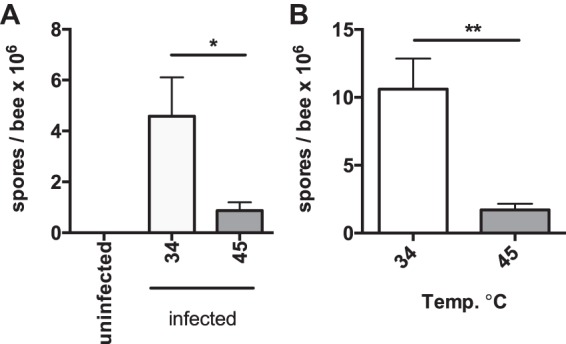
Heat shock reduces *N. ceranae* infection intensity in naturally or experimentally infected honey bees. (A) *N. ceranae* levels as determined by spore count using light microscopy in individual experimentally infected or control bees 8 days postinfection 24 h after exposure to 35° or 45°C for 4 h. (B) *N. ceranae* levels as determined by spore count using light microscopy in individual bees from an infected colony 24 h after exposure to 35° or 45°C for 4 h. Bars and error bars represent the mean ± standard error of the mean (SEM) for expression values of the genes of interest calculated using the ΔΔ*C_T_* method. Statistical significance is noted as *, *P* < 0.05; and **, *P* < 0.01.

We first determined how a heat shock protocol known to cause robust induction of the HSR in the honey bee (18) would impact *N. ceranae* infection. We experimentally infected bees and 7 days postinfection exposed the infected bees to 35° or 45°C for 4 h. After 24 additional h, we measured spore levels using light microscopy to determine the effects of heat shock on *N. ceranae* infection intensity. We found that maintaining bees (Fig. 1A) at 45°C for 4 h resulted in a dramatic reduction in spore levels. We found similar results for bees captured on the landing board of an infected colony and then maintained at 45°C for 4 h (Fig. 1B). We also found that this effect was dependent on the duration of the heat shock and temperature. There was no reduction in infection intensity in bees kept at 39°C even for 24 h (see Fig. S1A in the supplemental material). Bees had to be kept at 45°C for at least 2 h to reduce infection intensity (Fig. S1B). We found that a 4-h treatment of 45°C had no impact on honey bee survival during the course of the experiment (data not shown), in agreement with other studies on the thermotolerance of honey bees (19).

10.1128/mSphere.00219-19.1 FIG S1 (A) *N. ceranae* levels as determined by spore count using light microscopy in individual experimentally infected bees 8 days postinfection after exposure to 35°C or 39°C for 4 h or 39°C for 24 h. (B) *N. ceranae* levels as determined by spore count using light microscopy in individual experimentally infected bees 8 days postinfection 24 h after exposure to 45°C for 0, 1, 2, or 4 h. a ≠ b; *P* < 0.05; **, *P* < 0.01. Download FIG S1, DOCX file, 0.05 MB. Copyright © 2019 McNamara-Bordewick et al. 2019 McNamara-Bordewick et al. This content is distributed under the terms of the Creative Commons Attribution 4.0 International license.

### *N. ceranae* has lost the canonical HSF and possess a reduced set of core HSF1-dependent HSR target genes.

**FIG 2:**
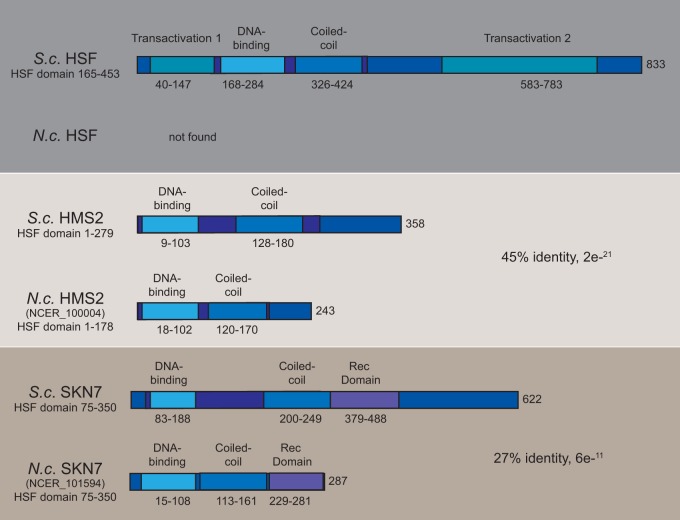
HSF, Skn7, and Hms2 in *N. ceranae* (*N.c.*) and S. cerevisiae (*S.c.*). A schematic representation of the domain structures of the HSF, SKN7, and HMS2 proteins from S. cerevisiae and the SKN7 and HMS2 proteins from *N. ceranae*. Shown are the location of the DNA-binding domain, the HSF domain, the coiled-coil domain, and known transactivation domains (based on references 39–41).

We examined the genome of *N. ceranae* to determine if the genome compaction found in microsporidia affects genes encoding the proteins involved in protecting cells from proteotoxic stress. Remarkably, our analysis of the *N. ceranae* genome revealed that this species has apparently lost the canonical HSF protein found in most eukaryotes, from fungi to animals and plants (20). *N. ceranae* possesses two genes encoding proteins with HSF-like domains, NCER_101594 and NCER_100004; however, alignment with fungal HSF proteins demonstrates that these proteins lack key activation domains (20) (Fig. 2 and S2). Based on the DNA-binding domain homology, NCER_101594 is most closely related to the response regulator SKN7 proteins of fungi (Fig. S2A). In fact, it possesses a receiver domain found in two-component signal proteins, such as SKN7 (Fig. S2B). Based on the DNA-binding domain homology, NCER_100004 is more closely related to HMS2. Examination of other available microsporidian genomes (21) reveals that all but one, Hamiltosporidium tvaerminnensis, have one or both of these two proteins (Table S1), but all apparently lack the canonical HSF. Equally striking is the apparent lack of *Msn2/4* gene homologs which encode stress-responsive transcription factors that regulate genes in response to diverse stresses, including thermal stress (2).

10.1128/mSphere.00219-19.2 FIG S2 (A) Alignment of the DNA-binding domains of HSF1-like proteins from *N. ceranae* (NCER_100004 [HMS2] and NCER_101594 [SKN7]), the DNA-binding domains of the SKN7 proteins from *Chaetomium thermophilium*, S. cerevisiae, S. pombe, and A. fumigatus, the DNA-binding domains of the HSF1 proteins from *C. thermophilium,* S. cerevisiae, S. pombe, A. fumigatus, and Homo sapiens, and the DNA-binding domains of the HMS2 protein of S. cerevisiae (based on Nakai [40]). (B) Alignment of the response regulator domain of the SKN7 proteins from *N. ceranae,* S. cerevisiae, S. pombe, and A. fumigatus, with the critical aspartate noted with an asterisk (based on Fassler and West [41]). Download FIG S2, DOCX file, 0.07 MB. Copyright © 2019 McNamara-Bordewick et al. 2019 McNamara-Bordewick et al. This content is distributed under the terms of the Creative Commons Attribution 4.0 International license.

10.1128/mSphere.00219-19.7 TABLE S1 HSF-like proteins in microsporidia genomes (sheet 1), Predicted cellular localization for microsporidia HSF-like proteins and proteins encoded by putative target genes (sheet 2), All predicted chaperone proteins in *N. ceranae* genome (sheet 3). Download Table S1, XLSX file, 0.02 MB. Copyright © 2019 McNamara-Bordewick et al. 2019 McNamara-Bordewick et al. This content is distributed under the terms of the Creative Commons Attribution 4.0 International license.

Recent work has identified a core set of HSF-dependent genes in the yeast Saccharomyces cerevisiae (22) and in mammals (21, 22). We used these to identify putative homologs (Table 1) and found that *N. ceranae* possesses apparent homologs for many of the proposed transcriptional targets of the pathway but has also lost a number of putative target genes, such that it only possess 7 of the 17 genes found to be core HSF-dependent genes in yeast. In addition, we identified other chaperone genes in the *N. ceranae* genome, including two HSP70 genes, NCER_101680 (predicted mitochondrial HSP70) and NCER_101994 (predicted to encode the HSP70 localized to the endoplasmic reticulum via a signal sequence [Table S1]), three additional HSP40 genes (NCER_101230, NCER_101367, and NCER_102171), and one gene for an HSP110, NCER_102035. A comparison between S. cerevisiae and *N. ceranae* reveals a sharp reduction in all classes of chaperones (Tables 2 and S1).

**TABLE 1:** *N. ceranae* homologs of HSF-like proteins and core HSF-dependent HSR genes identified in S. cerevisiae

S. cerevisiae gene name	*N. ceranae* homolog	Gene ID^*a*^	Proposed function^*b*^
*Hsf1*	Not found	NA	Transcription factor
*Skn7*	NCER_101594	9423197	Transcription factor
*Hms2*	NCER_100004	9423888	Transcription factor
*Msn2/4*	Not found	NA	Transcription factor
*Ssa2*	NCER_102023	9422542	HSP70
*Hsc82*	NCER_101194	9423122	HSP90
*Ydj1*	NCER_100860	9422122	HSP40
*Sis1*	NCER_101046	9424448	HSP40
*Hsp104*	NCER_101322	9424565	HSP104, disaggregase
*Cpr6*	NCER_101263	9424620	Cyclophilin
*Sti1*	NCER_101694	9422697	Cochaperone
*Mbf1*	Not found	NA	Transcriptional coactivator
*Btn2*	Not found	NA	Protein sorting
*Aha1*	Not found	NA	Cochaperone
*Hch1*	Not found	NA	Cochaperone
*Fes1*	Not found	NA	HSP70 NEF
*Cur1*	Not found	NA	Protein sorting
*Hsp42*	Not found	NA	HSP40
*Hsp78*	Not found	NA	HSP78, Mt disaggregase
*Hsp82*	Not found	NA	HSP90
*Mdj1*	Not found	NA	HSP40

a ID, identifier; NA, not applicable.

b NEF, nucleotide exchange factor; Mt, mitochondrial.

**TABLE 2:** Genes encoding chaperone proteins in the S. cerevisiae and *N. ceranae* genomes

Chaperone class	No. of genes (cytoplasmic, secretory, mitochondrial) in:	
	S. cerevisiae	*N. ceranae*
Hsp100	2 (1, 0, 1)	1 (1, 0, 0)
Hsp90	2 (2, 0, 0)	1 (1, 0, 0)
Hsp90 cochaperone	9 (9, 0, 0)	3 (3, 0, 0)
Hsp110	2 (1, 0, 0)	1 (1, 0, 0)
Hsp70	11 (6, 2, 3)	3 (1, 1, 1)
Hsp70 NEF	4 (2, 1, 1)	0 (0, 0, 0)
Hsp40/J	20 (13, 2, 5)	5 (4, 1, 0)
sSHP	3 (3, 0, 0)	0 (0, 0, 0)
Prefoldin	6 (6, 0, 0)	0 (0, 0, 0)
Chaperonin	10 (8, 0, 2)	8 (8, 0, 0)

### Heat shock induces a strong HSR in *N. ceranae*.

To determine whether *N. ceranae* could induce these genes in a heat shock response despite the loss of HSF, we experimentally infected bees and examined heat shock-dependent induction of putative heat shock genes for honey bee and *N. ceranae* in the midgut tissue of infected honey bees maintained at 35° or 45°C for 1 h at 8 days postinfection (Fig. S3). To use an unbiased approach to identify HSR target genes, we performed transcriptome profiling (RNA sequencing [RNA-seq]) of midguts from the bees. Six individual bee RNA-seq libraries (3 from each group) were prepared using the NEBNext Ultra RNA library preparation kit, and sequencing was then performed using an Illumina HiSeq platform. Transcriptome analysis of honey bee sequences revealed that HSR induction increased the expression of 283 genes compared to control bees and decreased the expression of 57 genes compared to control bees (Fig. 1 and S2A and Table S2). Transcriptome analysis of *N. ceranae* sequences revealed that HSR induction increased the expression of 147 genes compared to *N. ceranae* in control bees and decreased the expression of 100 genes compared to *N. ceranae* in control bees (Fig. 1 and S2B and Table S2).

10.1128/mSphere.00219-19.3 FIG S3 Volcano plot showing significantly upregulated (red) and downregulated (blue) genes for *A. mellifera* (A) and *N. ceranae* (B). Download FIG S3, DOCX file, 0.2 MB. Copyright © 2019 McNamara-Bordewick et al. 2019 McNamara-Bordewick et al. This content is distributed under the terms of the Creative Commons Attribution 4.0 International license.

10.1128/mSphere.00219-19.8 TABLE S2 Significant differential gene expression (Apis mellifera) (sheet 1), and significant differential gene expression (*N. ceranae*) (sheet 2). Download Table S2, XLSX file, 0.06 MB. Copyright © 2019 McNamara-Bordewick et al. 2019 McNamara-Bordewick et al. This content is distributed under the terms of the Creative Commons Attribution 4.0 International license.

To confirm the gene expression change observed by sequencing, we performed quantitative PCR (qPCR) on putative target genes, first in the honey bee. Relative to the β-actin gene, we observed robust induction of the homologs of the core HSF target genes, *Hsc70-4*, *Hsp70Ab*, *Hsp90*, *Hsp70Cb*, *Dnaja1*, and *Hspe1* in honey bees, which we had previously found to be induced by heat shock after 4 h (18) (Fig. S4). As expected, the transcriptional regulator *Hsf* was not induced after heat shock (Fig. S4), and β-actin gene levels were similar irrespective of temperature as assessed by threshold cycle (*C_T_*) values (Fig. S4), as shown before (18).

10.1128/mSphere.00219-19.4 FIG S4 Transcript levels of putative core honey bee HSR target genes *Hsc70-4*, (A) *Hsp70Ab* (B), *Hsp90* (C), *Hsp70Cb* (D), *Dnaja1* (E), *Hspe* (F), and Hsf (G) relative to β-actin (for which C_T_ values are shown in panel H) in the midgut of infected bees maintained for 1 h in cages at either 35°C or 45°C. Bars and error bars represent the mean ± standard error of the mean (SEM) for expression values of the genes of interest calculated using the ΔΔ*C_T_* method. Statistical significance is noted as *, *P* < 0.05; and **, *P* < 0.01. Download FIG S4, DOCX file, 0.2 MB. Copyright © 2019 McNamara-Bordewick et al. 2019 McNamara-Bordewick et al. This content is distributed under the terms of the Creative Commons Attribution 4.0 International license.

**FIG 3:**
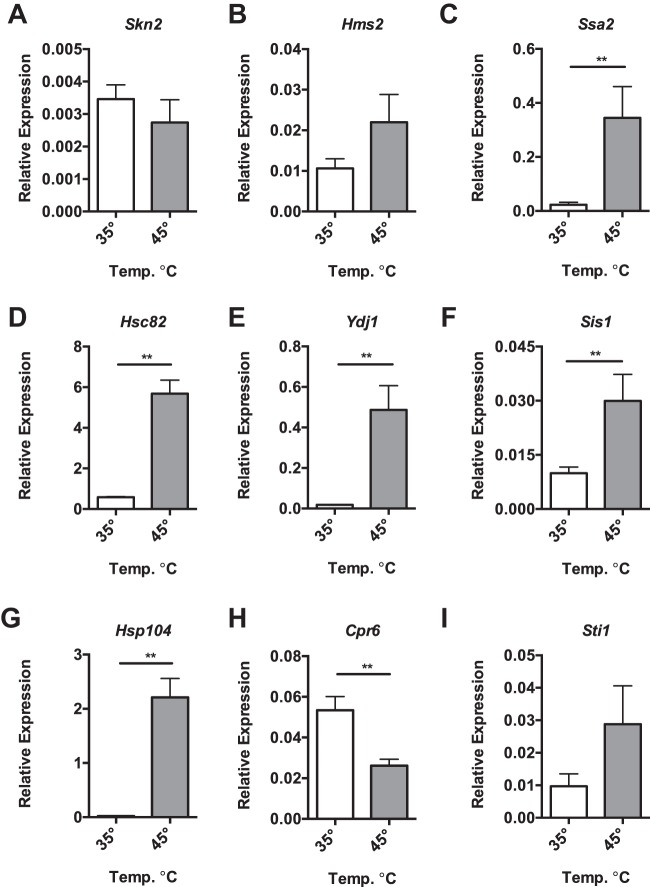
*N. ceranae* cells possess robust core response heat shock. (A to I) Shown are transcript levels of *N. ceranae* genes *Skn7* (NCER_101594) (A), *Hms2* (NCER_100004) (B), *Ssa1* (NCER_102023) (C), *Hsc82* (NCER_101194) (D), *Ydj1* (NCER_100860) (E), *Sis1* (NCER_101046) (F), *Hsp104* (NCER_101322) (G), *Cpr6* (NCER_101263) (H), and *Sti1* (NCER_101694) (I), relative to the *N. ceranae* β-actin gene in midguts from experimentally infected bees at 8 days postinfection after being maintained for 1 h in cages at either 35°C or 45°C. Bars and error bars represent the mean ± SEM for expression values of the genes of interest calculated using the ΔΔ*C_T_* method. Statistical significance is noted as *, *P* < 0.05; and **, *P* < 0.01.

**FIG 4:**
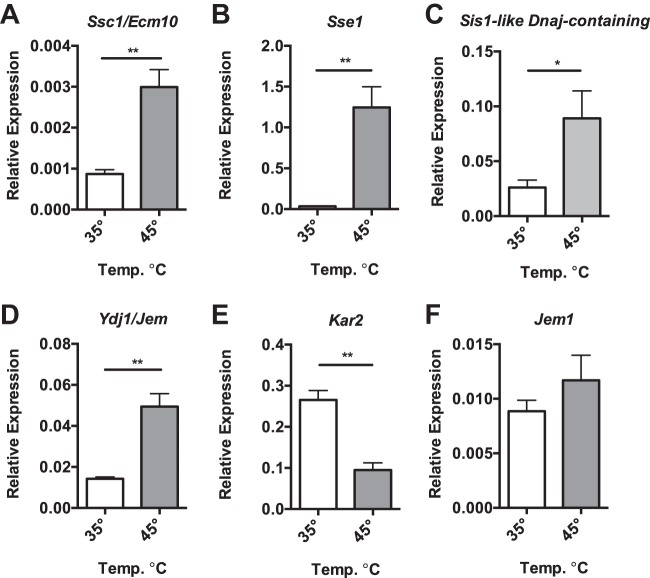
*N. ceranae* cells display strong broader response to heat shock. (A to F) Shown are transcript levels of *N. ceranae* genes *Ssc1/Ecm10* (NCER_101680) (A), *Sse1* (NCER_102035) (B) *Sis1-like Dnaj-containing* (NCER_102171) (C), *Ydj1/Jem* (NCER_101230) (D) *Kar2* (NCER_101994) (E), and *Jem1* (NCER_101367) (F) relative to the *N. ceranae* β-actin gene in midguts from experimentally infected bees at 8 days postinfection after being maintained for 1 h in cages at either 35°C or 45°C. Bars and error bars represent the mean ± SEM for expression values of the genes of interest calculated using the ΔΔ*C_T_* method. Statistical significance is noted as *, *P* < 0.05; and **, *P* < 0.01.

Then, we examined the transcript levels of the putative *N. ceranae* HSR genes. The HSF-like genes, *Skn7* (NCER_101594) (Fig. 3A) and *Hms2* (NCER_100004) (Fig. 3B), showed no difference in expression after heat shock. Measuring the genes encoding the core set of heat shock genes, we found increased expression of the genes encoding the HSP70 family member *Ssa1* (NCER_102023) (Fig. 3C), the HSP90 family member *Hsc82* (NCER_101194) (Fig. 3D), the HSP40 family members *Ydj1* (NCER_100860) (Fig. 3E) and *Sis1* (NCER_101046) (Fig. 3F), the disaggregase *Hsp104* (NCER_101322) (Fig. 3G), the cyclophilin *Cpr6* (NCER_101263) (Fig. 3H), and the cochaperone *Sti1* (NCER_101694) (Fig. 3I), relative to the *N. ceranae* β-actin gene. We also looked at the other chaperone proteins we found through homology searches and found that the HSP70 *Ssc1/Ecm10* (NCER_101680) (Fig. 4A), the HSP110 *Sse1* **(**NCER_102035) (Fig. 4B), the HSP40 *Sis1-like Dnaj-containing* (NCER_102171) (Fig. 4C), the HSP40 *Ydj1/Jem* (NCER_101230) (Fig. 4D), were all increased after heat shock relative to the *N. ceranae* β-actin gene. Interestingly, *Kar2* (NCER_101994) (Fig. 4E), encoding the predicted endoplasmic reticulum HSP70, was decreased in expression relative to *N. ceranae* β-actin gene after heat shock, while the HSP40 *Jem1* (NCER_101367), also predicted to have signal sequence, did not change after heat shock (Fig. 4F). (Table S3).

10.1128/mSphere.00219-19.9 TABLE S3 Differentially expressed genes associated with specific cellular processes (sheet 1), and top 20 differentially expressed genes (highlighted are those no known function) (sheet 2). Download Table S3, XLSX file, 0.02 MB. Copyright © 2019 McNamara-Bordewick et al. 2019 McNamara-Bordewick et al. This content is distributed under the terms of the Creative Commons Attribution 4.0 International license.

Interestingly, a recognizable heat shock element (HSE) was found in the promoters of a number of these genes, including NCER_102023, NCER_102035, NCER_101194, NCER_100860, and NCER_101322 (Fig. S5) but not NCER_101046, NCER_101263, NCER_102171, NCER_101230, NCER_101367, NCER_101994, NCER_101694, or NCER_101680 (data not shown).

10.1128/mSphere.00219-19.5 FIG S5 Heat shock elements (HSEs) in *N. ceranae* genes. Select HSR target genes with transcriptional start site, HSE, and core microsporidia transcriptional regulatory motifs are denoted. Download FIG S5, DOCX file, 0.02 MB. Copyright © 2019 McNamara-Bordewick et al. 2019 McNamara-Bordewick et al. This content is distributed under the terms of the Creative Commons Attribution 4.0 International license.

### Heat shock induces transcriptional shifts in the reduced metabolic pathways found in *N. ceranae*.

**FIG 5:**
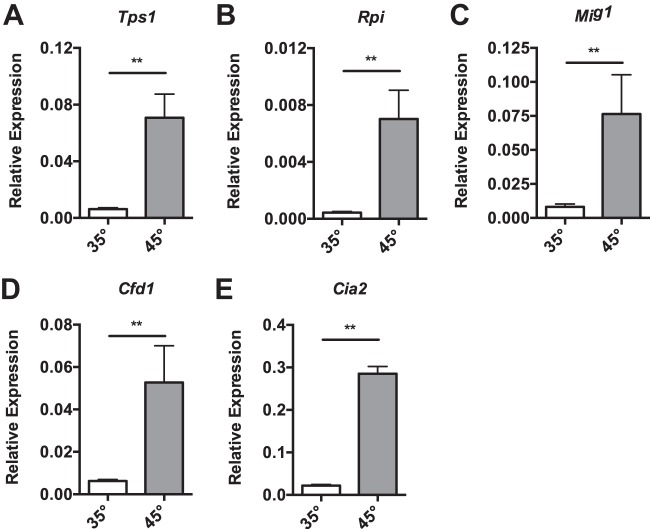
*N. ceranae* cells display metabolic changes in response to heat shock. (A to E) Shown are the transcript levels of *N. ceranae* genes *Tps1* (NCER_100278) (A), *Rpi* (NCER_102078) (B), *Mig1* (NCER_101715) (C), *Cfd1* (NCER_100723) (D), and *Cia2* (NCER_100612) (E) relative to the *N. ceranae* β-actin gene in midguts from experimentally infected bees at 8 days postinfection after being maintained for 1 h in cages at either 35°C or 45°C. Bars and error bars represent the mean ± SEM for expression values of the genes of interest calculated using the ΔΔ*C_T_* method. Statistical significance is noted as *, *P* < 0.05; and **, *P* < 0.01.

**FIG 6:**
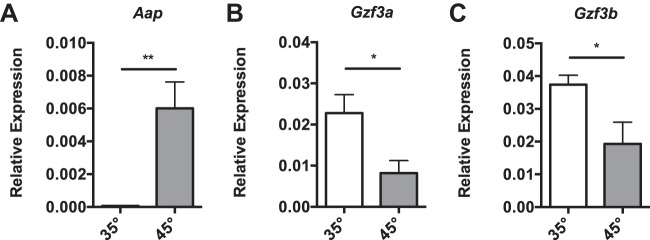
*N. ceranae* cells alter expression of amino acid metabolic genes in response to heat shock. (A to C) Shown are the transcript levels of *N. ceranae* genes *Aap* (NCER_102107) (A), *Gzf3a* (NCER_102253) (B), and *Gzf3b* (NCER_100170) (C) relative to the *N. ceranae* β-actin gene in midguts from experimentally infected bees at 8 days postinfection after being maintained for 1 h in cages at either 35°C or 45°C. Bars and error bars represent the mean ± SEM for expression values of the genes of interest calculated using the ΔΔ*C_T_* method. Statistical significance is noted as *, *P* < 0.05; and **, *P* < 0.01.

**FIG 7:**
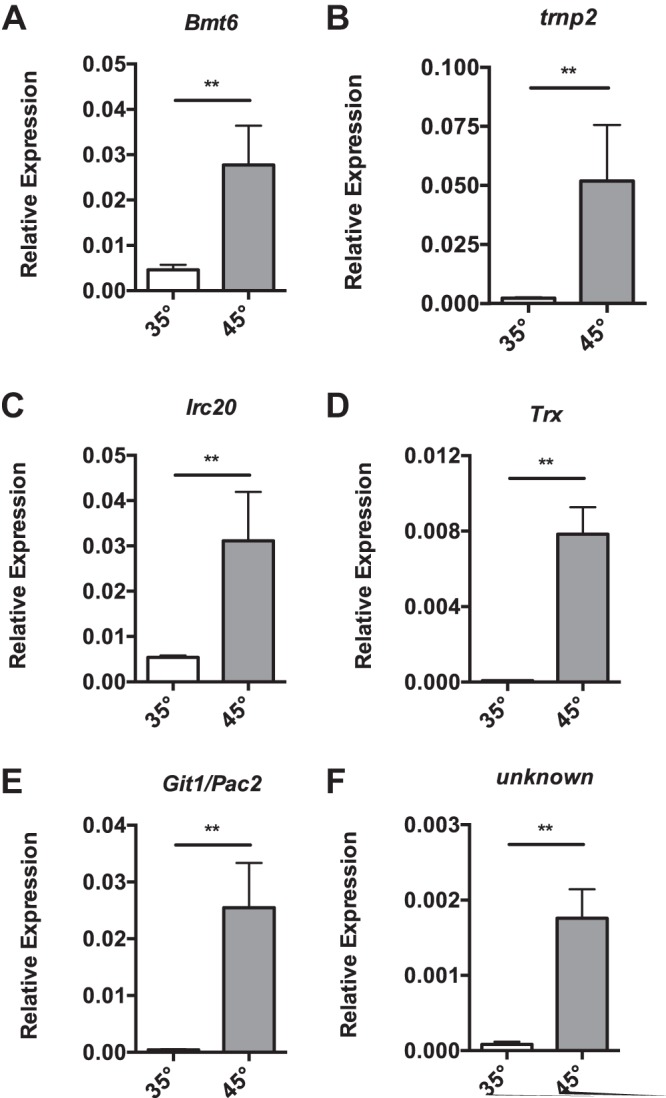
*N. ceranae* cells alter other cellular processes in response to heat shock. (A to F) Shown are the transcript levels of *N. ceranae* genes *Bmt6* (NCER_101100) (A), *trnp* (B), *Irc20* (NCER_100564) (C), *Trx* (NCER_100813) (D), *Git1/Pac2* (NCER_102041) (E), and a protein of unknown function (NCER_102300) (F) relative to the *N. ceranae* β-actin gene in midguts from experimentally infected bees at 8 days postinfection after being maintained for 1 h in cages at either 35° or 45°C. Bars and error bars represent the mean ± SEM for expression values of the genes of interest calculated using the ΔΔ*C_T_* method. Statistical significance is noted as *, *P* < 0.05; and **, *P* < 0.01.

In addition to alterations in protein folding machinery, we also observed that thermal stress leads to alterations in genes involved in diverse cellular processes (Table S3), and we used qPCR to validate a number of these gene changes (Fig. 5 to 7). Like other microsporidian species, *N. ceranae* possesses diminished metabolic pathways, missing key components found in most other eukaryotes. Heat shock is known to modulate metabolism in other fungal species, including S. cerevisiae (2), so we were especially interested in the impacts of thermal stress on the expression of *N. ceranae* metabolic genes. Interestingly, we found that some of the most highly induced genes after heat shock are involved in carbon and nitrogen metabolism. The trehalose phosphate synthase gene (*Tps1* [NCER_100278]), which encodes the key enzyme for making trehalose from glucose-6-phosphate, is transcriptionally upregulated (Fig. 5). Trehalose is a key osmoprotectant thought to be important for mediating resistance to thermal stress in yeast, although some evidence suggests that this protein has other roles in thermotolerance (23). Transcription of the ribose-5-phosphate isomerase gene (*Rpi* [NCER_102078]), which encodes the enzyme that catalyzes the conversion between ribose-5-phosphate (R5P) and ribulose-5-phosphate (Ru5P) as part of the nonoxidative phase of the pentose phosphate pathway (PPP), is highly upregulated. The two most important products from this process are the ribose-5-phosphate sugar used to make nucleotides, and NADPH, which is an important reducing agent for many cellular processes. R5P can be converted through a series of reactions to the glycolysis intermediates glyceraldehyde-3-phosphate and fructose-6-phosphate. However, microsporidia lack key enzymes for making nucleotides and instead take up nucleotides from the host cell (24), possibly suggesting that the primary function of the PPP in these species is to generate NADPH. We also found increased expression of the gene encoding MIG1 (*Mig1* [NCER_101715]), a regulatory factor involved in glucose repression of genes involved in the utilization of alternative carbon sources in S. cerevisiae (Fig. 5C). These results suggest that thermal stress remodels carbon metabolism to shunt glucose toward trehalose generation and production of reductive potential. *N. ceranae* under thermal stress also increases expression of the iron-sulfur cluster assembly protein genes *Cia2* (NCER_100612) (Fig. 5D) and *Cfd1* (NCER_100723) (Fig. 5D) (25), which help manufacture these cofactors critical for many metabolic enzymes. Iron sulfur cluster synthesis machinery is known to be preserved in microsporidia (26), and this process is split between the mitosome and the cytoplasm. Delivery of iron sulfur clusters to holoproteins has been shown to require an hsp70 chaperone system in most eukaryotes. In Saccharomyces cerevisiae, the Hsp70 SSQ1, its J protein cochaperone JAC1, and the nucleotide release factor MGE1 participate in this activity. *N. ceranae* does not have homologs to these proteins, although other hsp70 and associated factors that this species does possess could be involved in this assembly function.

We found that a number of genes thought to be involved in amino acid metabolism and procurement in yeast were upregulated by thermal stress in *N. ceranae* (Table S3). We found that two of the seven amino acid permeases (27) are upregulated by thermal stress (one of these, *Aap* [NCER_102107], is shown in Fig. 6A). The gene encoding glutamate synthase (*Glt* [NCER_101292]), which converts two glutamates to glutamine (and 2-oxoglutarate) while reducing NAD^+^ to NADH, was also upregulated by thermal stress. However, as microsporidia lack genes for generating most amino acids, the function of this gene in microsporidia is unclear. Most yeast species have a number of nitrogen-sensing pathways that allow them to respond to changes in the environment, including the nitrogen control repressible (NCR) pathway (28). The NCR pathway is regulated by a number of GATA factors (29). We found that *N. ceranae* possesses two GATA factors, *Gzf3a* (NCER_102253) and *Gzf3b* (NCER_100170), both of which are downregulated by heat shock (Fig. 6B and C).

### Heat shock in *N. ceranae* impacts diverse cellular processes at the transcriptional level.

In addition, we observed that thermal stress leads to alterations in many genes involved in other cellular processes, such as ribosome biogenesis and translation, DNA repair, and redox status (Table S3). The gene encoding 25S rRNA (uracil2843-N3)-methyltransferase (*Bmt6* [NCER_101100]), which is involved in ribosome biogenesis, is upregulated (Fig. 7A). The tRNA molecules with anticodons that specify proline inclusion in proteins by the ribosomes (tRNP) are highly upregulated by thermal stress (Fig. 7B). DNA repair genes were significantly upregulated after heat shock (Table S3) with the IRC20 ubiquitin ligase shown here (*Irc20* [NCER_100564]) (Fig. 7C). It is well appreciated that DNA repair is impaired after heat stress and that many organisms respond by increasing capacity (30). Interestingly, microsporidia are known to have reduced DNA repair machinery (31). Transcripts encoding a putative thioredoxin (*Trx* [NCER_100813]), which are often upregulated during oxidative stress (2), were also increased after heat shock (Fig. 7D).

We also observed that a number of genes were upregulated that share significant homology with transcription factors involved in differentiation in yeast (Table S3). *Git1/Pac2* (NCER_102041), most closely related to hypothetical protein YHR177W in S. cerevisiae, is upregulated (Fig. 7E). This protein contains a conserved Git1/Pac2 domain, identifying it as a member of the WOPR family. Transcription factors of this family are common to all fungi and control development, metabolism, and pathogenicity (32). Finally, heat shock induces a significant number of genes encoding proteins of unknown function. We found that only 81 of 167 upregulated genes had homologs outside microsporidia, and 11 of the top 20 induced genes encode proteins for which there is no known homolog (Table S3). For example, NCER_102300 is a protein of unknown function containing no known structural features, and it is the second most highly upregulated gene after heat shock (Table S3 and Fig. 7F).

## DISCUSSION

Fungal species display great variance in their tolerance to increased temperatures, but the majority prefer 12 to 30°C, while only a select few species are able to tolerate temperatures higher than 35°C (1). The existence of relatively few species able to grow at higher temperatures is thought to contribute to why mammals are fairly resistant to fungal infection. For other pathogenic fungi that can grow at mammalian body temperatures, the host organism can provide some buffering against environmental stresses (33). However, fungal species that are opportunistic pathogens, such as C. albicans, likely encounter substantial fluctuations in growth conditions and still possess robust cellular stress systems (3). As an obligate intracellular fungal pathogen, a microsporidian cell is able to take maximal advantage of the stable cellular environment of the host, which is maintained by the cellular and physiologic pathways designed to preserve cellular homeostasis. Our results mentioned above demonstrate that despite being obligate intracellular pathogens, microsporidia possess substantial ability to respond to thermal stress with transcriptional changes to typical HSR targets. In addition, we observe broad remodeling of gene expression affecting other diverse cellular processes. We found that some of the most highly induced genes after heat shock are involved in carbon and nitrogen metabolism. Thermal stress is known to have broad effects on metabolism in other fungal species, including Saccharomyces cerevisiae (reviewed in reference 2), Candida albicans (3), Schizosaccharomyces pombe (4), Aspergillus fumigatus (5), and Metarhizium anisopliae (6). Interestingly, microsporidia do not possess complete metabolic pathways as found in other fungi (13), instead obtaining the majority of metabolites from the host through an elaborated system of transporters (34). Our results suggest that despite these losses, microsporidia respond to thermal stress with remodeling of their metabolism through changes in their limited complement of metabolic genes and through changes in the expression of metabolite transport proteins, which are known to be important for nutrient uptake by microsporidia (34). In addition, we observed that thermal stress leads to alterations in many genes involved in other cellular processes, such as ribosome biogenesis and translation, DNA repair, and redox status. Yeast possess a shared response to diverse stresses known as the environmental stress response (ESR) (35, 36). Changes in genes involved in such diverse stress responses may be interpreted as evidence of an ESR in this species, as has been discovered in yeast (35, 36). However, if so, the mechanisms for regulating such a response remain obscure, as described below. In addition to known genes involved in other processes, 11 of the top 20 induced genes encode proteins for which there are no known homologs (Table S3). However, microsporidian genes are known to be highly divergent (34), and a high frequency of genes encoding proteins with no known function and containing domains solely found in other microsporidia has been observed in other studies of microsporidia (37). The apparent involvement of many of these genes in response to thermal stress is remarkable and warrants further study.

Perhaps the most interesting finding here is the apparent loss of the HSF transcription factor, which has been thought to be common to all eukaryotes (38). *N. ceranae* does possess two genes encoding proteins with HSF-like domains, NCER_101594 and NCER_100004. The proteins encoded by these genes appear to lack key activation domains (39) and are closer to SKN7 and HMS2, respectively. In both cases, the DNA-binding domain shares key structural domains with other fungal HSF-like proteins (40). It is interesting to speculate that one or both of these two proteins could function to modulate heat shock genes in the absence of HSF. SKN7 is known to have two main functions in other fungal species (41), one of which is to confer resistance to various cellular stresses, including oxidative, osmotic, and thermal stress. SKN7 is known to bind to HSE in other fungi, where it often coordinates gene expression with the transcription factor HSF1 (42). Interestingly, in these typical fungal species, SKN7 is part of a component signaling system with the partner protein SLN1 (43). However, microsporidian species, including *N. ceranae,* lack this protein. SKN7 has been implicated in stress responses in fungi (41, 44, 45). HMS2 was identified as a regulator of pseudohyphal differentiation in a screen that also identified SKN7 (46). It is also thought to be involved in responses to oxidative and thermal stress (47). In fact, it appears these two proteins arose as part of an ancient gene duplication event (48). Examination of other available microsporidian genomes (21) reveals that all but one, Hamiltosporidium tvaerminnensis, have one or both of these two proteins (Table S1), while all have lost the canonical HSF protein. The microsporidian cells exhibit a robust capacity for HSR gene induction despite this loss. Other eukaryotic pathogens appear to be missing the canonical HSF, including Toxoplasma gondii (49) and Plasmodium falciparum (50).

A recognizable HSE was only present in the promoters of a subset of the classical HSR genes found to be altered in expression, suggesting that other factors are likely involved in regulating these genes. The ESR is known to be dependent on MSN2/4 transcription factors (51). The apparent lack of *Msn2/4* gene homologs which encode these stress-responsive transcription factors in *N. ceranae* suggests that if there is a general stress system in this species, it is not regulated though means of these factors. We did observe that *N. ceranae* possesses the related transcription factor MIG1, which was highly upregulated after heat shock, making this protein another potential candidate for controlling the HSR in this species.

In addition to the loss of canonical stress regulators, there is a sharp reduction in the core HSF-dependent gene targets and other typical HSR-induced genes. Previous studies have found that microsporidia have reduced numbers of chaperone proteins, outside the TriC/CCT paralogs, which have been retained (52). Since microsporidia are intracellular pathogens, they capitalize on the stability of the host cell environment controlled by cellular and physiologic homeostatic pathways. Thus, we hypothesized that in the context of relaxed selection, the genomic reduction in microsporidia would affect genes involved in cellular stress responses. However, another possibility is that the reduced proteome complexity found in microsporidia led to a reduced need for proteostasis machinery. Some evidence suggests that the numbers of specific chaperones within a family (e.g., HSP40) scales with genome size (53).

Our data may provide some explanation of the seasonal prevalence and intensity of *Nosema* sp. infections in honey bees (54–56). It has been found that honey bees can recover from infection by *N. apis* when kept at the modestly elevated temperature of 37°C (57). However, previous work has shown that although honey bee temperatures during flight and endothermic shivering can be quite elevated, the increased heat is localized predominantly to the thorax, and the abdomen, which contains the infected midgut, does not experience dramatically higher temperatures (58). In fact, it appears that temperature increases normally experienced in the honey bee midgut are insufficient to affect *N. apis* infection (59). Honey bees are known to engage in a behavioral fever in some fungal infections (60) and prefer warmer temperatures when infected with *N. ceranae* (61). Thus, it would be interesting to determine whether these behaviors influence *Nosema* infection.

*N. ceranae* infection has traditionally been controlled by treatment with the drug fumagillin (62–65), a methionine aminopeptidase 2 inhibitor (MetAP2). However, the effectiveness of fumagillin treatment in controlling *N. ceranae* at the colony level has been shown to be limited in scope and duration (66). High doses of this drug are toxic to all eukaryotic cells (67), and, most critically, this drug is slated to become unavailable in the near future due to production problems. Thus, efforts to find alternative treatment strategies are critical to protect honey bees from *N. ceranae* infection. As a MetAP2, fumagillin works by interfering with protein synthesis and thereby disrupts proteostasis, the homeostasis of protein synthesis, folding, function, and degradation (68). As confirmed here, *Nosema* spp., both *N. apis* (69, 70) and *N. ceranae* (14, 15), exhibit an increased vulnerability to heat shock, another trigger of proteotoxic stress that works by denaturing proteins in the cytoplasm. It might be expected that an increased understanding of the sensitivity of *N. ceranae* to proteotoxic insult might identify novel treatment strategies for microsporidia infection in honey bees.

In summary, we corroborated the idea that *N. ceranae* demonstrates increased sensitivity to heat stress relative to its honey bee host. Characterization of the HSR in *N. ceranae* revealed microsporidian species have lost the transcriptional regulator HSF and retain an attenuated set of core HSF1-dependent HSR target genes. These remaining HSR target genes are strongly induced after heat shock, suggestive of a novel HSR regulation system. In addition to changes in proteostasis genes, thermal stress leads to expression changes in genes involved in diverse cellular processes, including metabolic pathways, ribosome biogenesis and translation, and DNA repair. These experiments represent the first steps aimed at understanding the cellular physiology of *N. ceranae* under different stress conditions. These findings may lead to novel therapeutic strategies aimed at controlling microsporidian infections impacting food production and human health.

## MATERIALS AND METHODS

### Honey bee tissue collection.

Honey bees were collected from the landing board of outbred colonies in New York, NY, consisting of a typical mix of Apis mellifera subspecies found in North America, at different times during the months of April to October. Only visibly healthy bees were collected, and all source colonies were visually inspected for symptoms of common bacterial, fungal, and viral diseases of honey bees. After cold anesthesia, bees were dissected, and tissues were set aside for gene expression analysis by storing in RNA*later* (Invitrogen, San Diego, CA).

### Ortholog screening of the *N. ceranae* and other microsporidian genomes.

HSR pathway and chaperone gene candidates from Saccharomyces cerevisiae (72) were used to find orthologs in the *N. ceranae* genome, as well as other available microsporidian genomes using the BLAST family of search functions (https://www.ncbi.nlm.nih.gov/), as described previously (73). The Kyoto Encyclopedia of Genes and Genomes (KEGG) database was also used as a guide for comparing pathways between species (74). For proteins of interest, we used the SignalP 4.1 server to predict the presence of a signal sequence, the TargetP 1.1 server to predict secretory or mitochondrial localization, and Prosite to predict the presence of an endoplasmic reticulum (ER) retention signal.

### Caged honey bee experiments.

For all caged experiments, honey bees were selected as described above and kept in 177.4 ml (6 oz.) square-bottomed *Drosophila* stock bottles (VWR) plugged with modified foam tube plugs (Jaece Industries). Bees were maintained in incubators at 35°C (unless otherwise stated) in the presence of PseudoQueen (Contech, Victoria, British Columbia, Canada) as a source of Queen mandibular pheromone (QMP) and used as per the manufacturer’s instructions. Bees were fed 33% sucrose via a modified 1.5-ml screw-cap tube. For heat shock, bees were fed 33% sucrose.

### Nosema ceranae infection and spore quantification.

*N. ceranae* spores were obtained from infected individuals for use in infection studies (64, 75). In addition, an isolate was obtained from this colony and serially passaged through bees, as performed previously (76). Spores from these bees were used in some experiments. To isolate spores, midguts from infected or uninfected bees were individually crushed in 0.5 ml H_2_O, and the spore number was assessed by light microscopy. Midguts were washed 3 times with water and resuspended in 33% sucrose syrup at a concentration of 1 × 10^6^ spores per ml. Bees were allowed to consume food *ad libitum* for 24 h before food was replaced with 33% sucrose syrup alone. Bees in the uninfected group always received sucrose syrup containing a midgut from an uninfected bee processed in the same way as the midgut-containing spores. Infection intensity was also quantified by counting number of spores per bee using light microscopy (77).

### RNA isolation, reverse transcription, and quantitative PCR for gene expression analysis.

Midguts were used for gene expression analysis. All dissected material was placed into RNA*later* (Invitrogen, San Diego, CA) for storage prior to analysis of individual workers’ gene expression. RNA was prepared from bees from the described populations by manually crushing the tissue of interest with a disposable pestle in TRIzol reagent (Invitrogen) and extracting the RNA as per the manufacturer’s instructions. RNA was subsequently DNase I treated by RQ1 RNase-free DNase (Promega, Madison, WI) and quantified. cDNA was synthesized using approximately 1 μg of RNA with the iScript cDNA synthesis kit (Bio-Rad, Hercules, CA). Typically, 1 μl of cDNA was then used as a template for quantitative PCR to determine the levels of expression of genes of interest using the iQ SYBR green supermix (Bio-Rad) in an iCycler thermocycler (Bio-Rad). Primer sequences for transcripts of genes of interest developed for this study are in Table S4. For honey bee genes, the difference between the threshold cycle (*C_T_*) number for the honey bee β-actin gene and that of the gene of interest was used to calculate expression levels relative to the β-actin gene using the ΔΔ*C_T_* method. For infection levels, the difference between the *C_T_* number for the honey bee β-actin gene and that of the primers for the *N. ceranae* β-actin gene was used with the ΔΔ*C_T_* method. A sample was considered negative for a specific *Nosema* species if it did not amplify any product by 35 cycles, and zero was entered as the value in these cases. For *N. ceranae* genes, the difference between the *C_T_* number for *N. ceranae* β-actin and that of the gene of interest was used to calculate expression levels relative to *N. ceranae* β-actin using the ΔΔ*C_T_* method.

10.1128/mSphere.00219-19.10 TABLE S4 Primer sequences developed for use in this study. Download Table S4, XLSX file, 0.01 MB. Copyright © 2019 McNamara-Bordewick et al. 2019 McNamara-Bordewick et al. This content is distributed under the terms of the Creative Commons Attribution 4.0 International license.

### RNA-seq.

For RNA-seq analysis, RNA was isolated from midgut samples as described above. After quantifying and checking the purity of RNA, it was shipped to GENEWIZ (South Plainfield, NJ, USA). RNA was submitted to additional quality control analysis before mRNA was enriched by poly(A) selection for library preparation using a NEBNext Ultra RNA library preparation kit. Sequencing was then performed using an Illumina HiSeq platform. Sequence reads were trimmed to remove possible adapter sequences and nucleotides with poor quality using Trimmomatic v.0.36. The reads were then mapped to the *A. mellifera* and *N. ceranae* reference genomes available on NCBI using the STAR aligner. The RNA-seq aligner is executed using a splice aligner, which detects splice junctions and incorporating them to help align the entire read sequences. BAM files were generated as a result of this step. Gene hit counts were calculated using Feature counts from the Subread package. The statistics of the raw sequence data and mapping of the reads to the reference genome are found in Table S2. After mapping and total gene hit count calculation, the total gene hit count table was used for downstream differential expression analysis using DESeq2. Genes with an adjusted *P* value of <0.05 and absolute log_2_ fold change of >1 were called significant differentially expressed genes (DEGs) for each comparison. Volcano plot analysis shows the global transcriptional change between groups. All the genes are plotted, and each data point represents a gene (Fig. S3). The log_2_ fold change of each gene is represented on the *x* axis, and the log_10_ *P* value is shown on the *y* axis. An adjusted *P* value of 0.05 and a log_2_ fold change of 1 are indicated by red dots. These represent upregulated genes. An adjusted *P* value of 0.05 and a log_2_ fold change of −1 are indicated by blue dots. These represent downregulated genes. To further investigate the function of the DEGs, we used a resource kindly provided by Michelle Flenniken (78) in which they used reciprocal BLAST+ (79) to identify the Drosophila melanogaster orthologs and homologs of all honey bee genes (80). Biological process (BP) functional enrichment analysis and Gene Ontology enrichment analysis were performed with DAVID using both the honey bee gene lists and the lists of the corresponding fruit fly homologs (81, 82).

### Statistical analysis.

For analysis, data were log_10_ transformed and compared using unpaired *t* tests with Welch’s correction when values fit normal distributions or Mann-Whitney U nonparametric tests when they did not fit normal distributions. Normality was assessed using Shapiro-Wilk tests. When more than two groups were being compared, data were compared using one-way analysis of variance (ANOVA) with Tukey’s multiple-comparison test when values fit normal distributions or a Kruskal-Wallis test.

### Accession number(s).

The RNA sequence information in this study has been submitted to the Gene Expression Omnibus database under accession number GSE128364.

10.1128/mSphere.00219-19.6 FIG S6 Diagram of components of the core HSR pathway conserved in honey bees and Nosema ceranae. Download FIG S6, DOCX file, 0.06 MB. Copyright © 2019 McNamara-Bordewick et al. 2019 McNamara-Bordewick et al. This content is distributed under the terms of the Creative Commons Attribution 4.0 International license.

## References

[1] Robert VA, Casadevall A 2009 Vertebrate endothermy restricts most fungi as potential pathogens. J Infect Dis 200:1623–1626. doi:10.1086/644642. 19827944

[2] Morano KA, Grant CM, Moye-Rowley WS 2012 The response to heat shock and oxidative stress in Saccharomyces cerevisiae. Genetics 190:1157–1195. doi:10.1534/genetics.111.128033. 22209905 PMC3316637

[3] Brown AJP, Budge S, Kaloriti D, Tillmann A, Jacobsen MD, Yin Z, Ene IV, Bohovych I, Sandai D, Kastora S, Potrykus J, Ballou ER, Childers DS, Shahana S, Leach MD 2014 Stress adaptation in a pathogenic fungus. J Exp Biol 217:144–155. doi:10.1242/jeb.088930. 24353214 PMC3867497

[4] Glatz A, Pilbat A-M, Németh GL, Vince-Kontár K, Jósvay K, Hunya Á, Udvardy A, Gombos I, Péter M, Balogh G, Horváth I, Vígh L, Török Z 2016 Involvement of small heat shock proteins, trehalose, and lipids in the thermal stress management in Schizosaccharomyces pombe. Cell Stress Chaperones 21:327–338. doi:10.1007/s12192-015-0662-4. 26631139 PMC4786532

[5] Albrecht D, Guthke R, Brakhage AA, Kniemeyer O 2010 Integrative analysis of the heat shock response in Aspergillus fumigatus. BMC Genomics 11:32. doi:10.1186/1471-2164-11-32. 20074381 PMC2820008

[6] Wang Z-X, Zhou X-Z, Meng H-M, Liu Y-J, Zhou Q, Huang B 2014 Comparative transcriptomic analysis of the heat stress response in the filamentous fungus Metarhizium anisopliae using RNA-seq. Appl Microbiol Biotechnol 98:5589–5597. doi:10.1007/s00253-014-5763-y. 24769907

[7] Leach MD, Cowen LE 2014 To sense or die: mechanisms of temperature sensing in fungal pathogens. Curr Fungal Infect Rep 8:185–191. doi:10.1007/s12281-014-0182-1.

[8] Weiss LM, Becnel JJ (ed). 2014 Microsporidia: pathogens of opportunity, 1st ed. John Wiley & Sons Inc, Hoboken, NJ.

[9] Reference deleted.

[10] Martín-Hernández R, Bartolomé C, Chejanovsky N, Le Conte Y, Dalmon A, Dussaubat C, García-Palencia P, Meana A, Pinto MA, Soroker V, Higes M 2018 Nosema ceranaein Apis mellifera: a 12 years postdetection perspective. Environ Microbiol 20:1302–1329. doi:10.1111/1462-2920.14103. 29575513

[11] Goblirsch M 2017 *Nosema ceranae* disease of the honey bee (*Apis mellifera*). Apidologie 49:131–150. doi:10.1007/s13592-017-0535-1.

[12] Evans JD, Schwarz RS 2011 Bees brought to their knees: microbes affecting honey bee health. Trends Microbiol 19:614–620. doi:10.1016/j.tim.2011.09.003. 22032828

[13] Nakjang S, Williams TA, Heinz E, Watson AK, Foster PG, Sendra KM, Heaps SE, Hirt RP, Martin Embley T 2013 Reduction and expansion in microsporidian genome evolution: new insights from comparative genomics. Genome Biol Evol 5:2285–2303. doi:10.1093/gbe/evt184. 24259309 PMC3879972

[14] Martín-Hernández R, Meana A, García-Palencia P, Marín P, Botías C, Garrido-Bailón E, Barrios L, Higes M 2009 Effect of temperature on the biotic potential of honeybee microsporidia. Appl Environ Microbiol 75:2554–2557. doi:10.1128/AEM.02908-08. 19233948 PMC2675226

[15] Higes M, García-Palencia P, Botías C, Meana A, Martín-Hernández R 2010 The differential development of microsporidia infecting worker honey bee (Apis mellifera) at increasing incubation temperature. Environ Microbiol Rep 2:745–748. doi:10.1111/j.1758-2229.2010.00170.x. 23766279

[16] Vabulas RM, Raychaudhuri S, Hayer-Hartl M, Hartl FU 2010 Protein folding in the cytoplasm and the heat shock response. Cold Spring Harb Perspect Biol 2:a004390. doi:10.1101/cshperspect.a004390. 21123396 PMC2982175

[17] Morimoto RI 2011 The heat shock response: systems biology of proteotoxic stress in aging and disease. Cold Spring Harb Symp Quant Biol 76:91–99. doi:10.1101/sqb.2012.76.010637. 22371371

[18] McKinstry M, Chung C, Truong H, Johnston BA, Snow JW 2017 The heat shock response and humoral immune response are mutually antagonistic in honey bees. Sci Rep 7:8850. doi:10.1038/s41598-017-09159-4. 28821863 PMC5562734

[19] Kovac H, Käfer H, Stabentheiner A, Costa C 2014 Metabolism and upper thermal limits of Apis mellifera carnica and A. m. ligustica. Apidologie 45:664–677. doi:10.1007/s13592-014-0284-3. 25378763 PMC4218932

[20] Joutsen J, Sistonen L 2019 Tailoring of proteostasis networks with heat shock factors. Cold Spring Harb Perspect Biol 11:a034066. doi:10.1101/cshperspect.a034066. 30420555 PMC6442201

[21] Mahat DB, Salamanca HH, Duarte FM, Danko CG, Lis JT 2016 Mammalian heat shock response and mechanisms underlying its genome-wide transcriptional regulation. Mol Cell 62:63–78. doi:10.1016/j.molcel.2016.02.025. 27052732 PMC4826300

[22] Solís EJ, Pandey JP, Zheng X, Jin DX, Gupta PB, Airoldi EM, Pincus D, Denic V 2016 Defining the essential function of yeast Hsf1 reveals a compact transcriptional program for maintaining eukaryotic proteostasis. Mol Cell 63:60–71. doi:10.1016/j.molcel.2016.05.014. 27320198 PMC4938784

[23] Pallapati AR, Das E, Roy I 2017 Crosstalk between osmolytes and cellular chaperones: examples in Saccharomyces cerevisiae, p 55–75. *In* Rajendrakumar L, Dar TA (ed), Cellular osmolytes. Springer Singapore, Singapore.

[24] Dean P, Hirt RP, Embley TM 2016 Microsporidia: why make nucleotides if you can steal them? PLoS Pathog 12:e1005870. doi:10.1371/journal.ppat.1005870. 27855212 PMC5113988

[25] Lill R 2009 Function and biogenesis of iron-sulphur proteins. Nature 460:831–838. doi:10.1038/nature08301. 19675643

[26] Freibert S-A, Goldberg AV, Hacker C, Molik S, Dean P, Williams TA, Nakjang S, Long S, Sendra K, Bill E, Heinz E, Hirt RP, Lucocq JM, Embley TM, Lill R 2016 Evolutionary conservation and in vitro reconstitution of microsporidian iron-sulfur cluster biosynthesis. Nat Commun 8:13932. doi:10.1038/ncomms13932. PMC5216125 28051091

[27] Chetia H, Kabiraj D, Sharma S, Bora U 2017 Comparative insights to the transportome of Nosema: a genus of parasitic microsporidians. bioRxiv doi:10.1101/110809.

[28] Ljungdahl PO, Daignan-Fornier B 2012 Regulation of amino acid, nucleotide, and phosphate metabolism in Saccharomyces cerevisiae. Genetics 190:885–929. doi:10.1534/genetics.111.133306. 22419079 PMC3296254

[29] Milias-Argeitis A, Oliveira AP, Gerosa L, Falter L, Sauer U, Lygeros J 2016 Elucidation of genetic interactions in the yeast GATA-factor network using Bayesian model selection. PLoS Comput Biol 12:e1004784. doi:10.1371/journal.pcbi.1004784. 26967983 PMC4788432

[30] Oei AL, Vriend LEM, Crezee J, Franken NAP, Krawczyk PM 2015 Effects of hyperthermia on DNA repair pathways: one treatment to inhibit them all. Radiat Oncol 10:165. doi:10.1186/s13014-015-0462-0. 26245485 PMC4554295

[31] Gill EE, Fast NM 2007 Stripped-down DNA repair in a highly reduced parasite. BMC Mol Biol 8:24. doi:10.1186/1471-2199-8-24. 17374165 PMC1851970

[32] Lohse MB, Rosenberg OS, Cox JS, Stroud RM, Finer-Moore JS, Johnson AD 2014 Structure of a new DNA-binding domain which regulates pathogenesis in a wide variety of fungi. Proc Natl Acad Sci U S A 111:10404–10410. doi:10.1073/pnas.1410110111. 24994900 PMC4115540

[33] Leach MD, Cowen LE 2013 Surviving the heat of the moment: a fungal pathogens perspective. PLoS Pathog 9:e1003163. doi:10.1371/journal.ppat.1003163. 23505364 PMC3591304

[34] Dean P, Sendra KM, Williams TA, Watson AK, Major P, Nakjang S, Kozhevnikova E, Goldberg AV, Kunji ERS, Hirt RP, Embley TM 2018 Transporter gene acquisition and innovation in the evolution of Microsporidia intracellular parasites. Nat Commun 9:1709. doi:10.1038/s41467-018-03923-4. 29703975 PMC5923384

[35] Gasch AP, Spellman PT, Kao CM, Carmel-Harel O, Eisen MB, Storz G, Botstein D, Brown PO 2000 Genomic expression programs in the response of yeast cells to environmental changes. Mol Biol Cell 11:4241–4257. doi:10.1091/mbc.11.12.4241. 11102521 PMC15070

[36] Causton HC, Ren B, Koh SS, Harbison CT, Kanin E, Jennings EG, Lee TI, True HL, Lander ES, Young RA 2001 Remodeling of yeast genome expression in response to environmental changes. Mol Biol Cell 12:323–337. doi:10.1091/mbc.12.2.323. 11179418 PMC30946

[37] Reinke AW, Balla KM, Bennett EJ, Troemel ER 2017 Identification of microsporidia host-exposed proteins reveals a repertoire of rapidly evolving proteins. Nat Commun 8:14023. doi:10.1038/ncomms14023. 28067236 PMC5423893

[38] Åkerfelt M, Morimoto RI, Sistonen L 2010 Heat shock factors: integrators of cell stress, development and lifespan. Nat Rev Mol Cell Biol 11:545–555. doi:10.1038/nrm2938. 20628411 PMC3402356

[39] Veri AO, Robbins N, Cowen LE 2018 Regulation of the heat shock transcription factor Hsf1 in fungi: implications for temperature-dependent virulence traits. FEMS Yeast Res 18:foy041. doi:10.1093/femsyr/foy041. PMC7190891 29788061

[40] Nakai A 2016 News and views. Nat Struct Mol Biol 23:93–95. doi:10.1038/nsmb.3165. 26840894

[41] Fassler JS, West AH 2011 Fungal Skn7 stress responses and their relationship to virulence. Eukaryot Cell 10:156–167. doi:10.1128/EC.00245-10. 21131436 PMC3067409

[42] Raitt DC, Johnson AL, Erkine AM, Makino K, Morgan B, Gross DS, Johnston LH 2000 The Skn7 response regulator of Saccharomyces cerevisiae interacts with Hsf1 in vivo and is required for the induction of heat shock genes by oxidative stress. Mol Biol Cell 11:2335–2347. doi:10.1091/mbc.11.7.2335. 10888672 PMC14923

[43] Schaller GE, Shiu S-H, Armitage JP 2011 Two-component systems and their co-option review for eukaryotic signal transduction. Curr Biol 21:R320–R330. doi:10.1016/j.cub.2011.02.045. 21549954

[44] Hussain M, Hamid MI, Wang N, Bin L, Xiang M, Liu X 2016 The transcription factor SKN7 regulates conidiation, thermotolerance, apoptotic-like cell death and parasitism in the nematode endoparasitic fungus Hirsutella minnesotensis. Sci Rep 6:30047. doi:10.1038/srep30047. 27436205 PMC4951753

[45] Wormley FL, Heinrich G, Miller JL, Perfect JR, Cox GM 2005 Identification and characterization of an SKN7 homologue in Cryptococcus neoformans. Infect Immun 73:5022–5030. doi:10.1128/IAI.73.8.5022-5030.2005. 16041017 PMC1201254

[46] Lorenz MC, Heitman J 1998 Regulators of pseudohyphal differentiation in Saccharomyces cerevisiae identified through multicopy suppressor analysis in ammonium permease mutant strains. Genetics 150:1443–1457. 9832522 10.1093/genetics/150.4.1443 PMC1460428

[47] Ayer A, Fellermeier S, Fife C, Li SS, Smits G, Meyer AJ, Dawes IW, Perrone GG 2012 A genome-wide screen in yeast identifies specific oxidative stress genes required for the maintenance of sub-cellular redox homeostasis. PLoS One 7:e44278. doi:10.1371/journal.pone.0044278. 22970195 PMC3435413

[48] Kellis M, Birren BW, Lander ES 2004 Proof and evolutionary analysis of ancient genome duplication in the yeast Saccharomyces cerevisiae. Nature 428:617–624. doi:10.1038/nature02424. 15004568

[49] Ma YF, Zhang Y, Kim K, Weiss LM 2004 Identification and characterisation of a regulatory region in the Toxoplasma gondii hsp70 genomic locus. Int J Parasitol 34:333–346. doi:10.1016/j.ijpara.2003.11.020. 15003494 PMC3109639

[50] Sayeed SK, Shah V, Chaubey S, Singh M, Alampalli SV, Tatu US 2014 Identification of heat shock factor binding protein in Plasmodium falciparum. Malar J 13:118. doi:10.1186/1475-2875-13-118. 24674379 PMC3994269

[51] Roetzer A, Gregori C, Jennings AM, Quintin J, Ferrandon D, Butler G, Kuchler K, Ammerer G, Schüller C 2008 Candida glabrataenvironmental stress response involves Saccharomyces cerevisiaeMsn2/4 orthologous transcription factors. Mol Microbiol 69:603–620. doi:10.1111/j.1365-2958.2008.06301.x. 18547390 PMC2610386

[52] Heinz E, Williams TA, Nakjang S, Noël CJ, Swan DC, Goldberg AV, Harris SR, Weinmaier T, Markert S, Becher D, Bernhardt J, Dagan T, Hacker C, Lucocq JM, Schweder T, Rattei T, Hall N, Hirt RP, Embley TM 2012 The genome of the obligate intracellular parasite Trachipleistophora hominis: new insights into microsporidian genome dynamics and reductive evolution. PLoS Pathog 8:e1002979. doi:10.1371/journal.ppat.1002979. 23133373 PMC3486916

[53] Powers ET, Balch WE 2013 Diversity in the origins of proteostasis networks—a driver for protein function in evolution. Nat Rev Mol Cell Biol 14:237–248. doi:10.1038/nrm3542. 23463216 PMC3718298

[54] Runckel C, Flenniken ML, Engel JC, Ruby JG, Ganem D, Andino R, DeRisi JL 2011 Temporal analysis of the honey bee microbiome reveals four novel viruses and seasonal prevalence of known viruses, Nosema, and Crithidia. PLoS One 6:e20656. doi:10.1371/journal.pone.0020656. 21687739 PMC3110205

[55] Traver BE, Williams MR, Fell RD 2012 Comparison of within hive sampling and seasonal activity of Nosema ceranae in honey bee colonies. J Invertebr Pathol 109:187–193. doi:10.1016/j.jip.2011.11.001. 22085836

[56] Chen Y-W, Chung W-P, Wang C-H, Solter LF, Huang W-F 2012 Nosema ceranae infection intensity highly correlates with temperature. J Invertebr Pathol 111:264–267. doi:10.1016/j.jip.2012.08.014. 22982233

[57] Lotmar R 1943 Über den Einfluss der Temperatur auf den Parasiten Nosema apis. Beih Schweiz Bienen-Ztg 1:261–284.

[58] Stabentheiner A, Kovac H, Brodschneider R 2010 Honeybee colony thermoregulation–regulatory mechanisms and contribution of individuals in dependence on age, location and thermal stress. PLoS One 5:e8967. doi:10.1371/journal.pone.0008967. 20126462 PMC2813292

[59] Bailey L 1959 The natural mechanism of suppression of Nosema apis Zander in enzootically infected colonies of the honey bee, Apis mellifera Linnaeus. J Insect Pathol 1:347–350.

[60] Starks P, Blackie C, Seeley T 2000 Fever in honeybee colonies. Naturwissenschaften 87:229–231. doi:10.1007/s001140050709. 10883439

[61] Campbell J, Kessler B, Mayack C, Naug D 2010 Behavioural fever in infected honeybees: parasitic manipulation or coincidental benefit? Parasitology 137:1487–1491. doi:10.1017/S0031182010000235. 20500914

[62] Higes M, Martín R, Meana A 2006 Nosema ceranae, a new microsporidian parasite in honeybees in Europe. J Invertebr Pathol 92:93–95. doi:10.1016/j.jip.2006.02.005. 16574143

[63] Higes M, Nozal MJ, Alvaro A, Barrios L, Meana A, Martín-Hernández R, Bernal JL, Bernal J 2011 The stability and effectiveness of fumagillin in controlling Nosema ceranae (Microsporidia) infection in honey bees (Apis mellifera) under laboratory and field conditions. Apidologie 42:364–377. doi:10.1007/s13592-011-0003-2.

[64] Huang W-F, Jiang J-H, Chen Y-W, Wang C-H 2007 A Nosema ceranae isolate from the honeybee Apis mellifera. Apidologie 38:30–37. doi:10.1051/apido:2006054.

[65] Chen YP, Huang ZY 2010 Nosema ceranae, a newly identified pathogen of Apis mellifera in the USA and Asia. Apidologie 41:364–374. doi:10.1051/apido/2010021.

[66] Mendoza Y, Diaz-Cetti S, Ramallo G, Santos E, Porrini M, Invernizzi C 2016 Nosema ceranae winter control: study of the effectiveness of different fumagillin treatments and consequences on the strength of honey bee (Hymenoptera: Apidae) colonies. J Econ Entomol 110:1–5. doi:10.1093/jee/tow228. 28025388

[67] Huang W-F, Solter LF, Yau PM, Imai BS 2013 Nosema ceranae escapes fumagillin control in honey bees. PLoS Pathog 9:e1003185. doi:10.1371/journal.ppat.1003185. 23505365 PMC3591333

[68] Taylor RC, Berendzen KM, Dillin A 2014 Systemic stress signalling: understanding the cell non-autonomous control of proteostasis. Nat Rev Mol Cell Biol 15:211–217. doi:10.1038/nrm3752. 24556842 PMC5922984

[69] Burnside CF, Revell IL 1948 Observations on nosema disease of honey bees. J Econ Entomol 41:603–607. doi:10.1093/jee/41.4.603.

[70] Woyciechowski M, Czekońska K 1999 The effect of temperature on Nosema apisZander (Microsporida, Nosematidae) infection in honey bees (Apis mellifera). Parasite 6:185–187. doi:10.1051/parasite/1999062185.

[71] Reference deleted.

[72] Verghese J, Abrams J, Wang Y, Morano KA 2012 Biology of the heat shock response and protein chaperones: budding yeast (Saccharomyces cerevisiae) as a model system. Microbiol Mol Biol Rev 76:115–158. doi:10.1128/MMBR.05018-11. 22688810 PMC3372250

[73] Johnston BA, Hooks KB, McKinstry M, Snow JW 2016 Divergent forms of endoplasmic reticulum stress trigger a robust unfolded protein response in honey bees. J Insect Physiol 86:1–10. doi:10.1016/j.jinsphys.2015.12.004. 26699660

[74] Kanehisa M, Goto S 2000 KEGG: Kyoto Encyclopedia of Genes and Genomes. Nucleic Acids Res 28:27–30. doi:10.1093/nar/28.1.27. 10592173 PMC102409

[75] Higes M, García-Palencia P, Martín-Hernández R, Meana A 2007 Experimental infection of Apis mellifera honeybees with Nosema ceranae (Microsporidia). J Invertebr Pathol 94:211–217. doi:10.1016/j.jip.2006.11.001. 17217954

[76] Holt HL, Aronstein KA, Grozinger CM 2013 Chronic parasitization by Nosema microsporidia causes global expression changes in core nutritional, metabolic and behavioral pathways in honey bee workers (Apis mellifera). BMC Genomics 14:799. doi:10.1186/1471-2164-14-799. 24245482 PMC4046765

[77] Cantwell GE 1970 Standard methods for counting Nosema spores. Am Bee J 110:222–223.

[78] Brutscher LM, Daughenbaugh KF, Flenniken ML 2017 Virus and dsRNA-triggered transcriptional responses reveal key components of honey bee antiviral defense. Sci Rep 7:6448. doi:10.1038/s41598-017-06623-z. 28743868 PMC5526946

[79] Camacho C, Coulouris G, Avagyan V, Ma N, Papadopoulos J, Bealer K, Madden TL 2009 BLAST+: architecture and applications. BMC Bioinformatics 10:421–429. doi:10.1186/1471-2105-10-421. 20003500 PMC2803857

[80] Elsik CG, Worley KC, Bennett AK, Beye M, Camara F, Childers CP, de Graaf DC, Debyser G, Deng J, Devreese B, Elhaik E, Evans JD, Foster LJ, Graur D, Guigó R, Hoff KJ, Holder ME, Hudson ME, Hunt GJ, Jiang H, Joshi V, Khetani RS, Kosarev P, Kovar CL, Ma J, Maleszka R, Moritz RFA, Munoz-Torres MC, Murphy TD, Muzny DM, Newsham IF, Reese JT, Robertson HM, Robinson GE, Rueppell O, Solovyev V, Stanke M, Stolle E, Tsuruda JM, Van Vaerenbergh M, Waterhouse RM, Weaver DB, Whitfield CW, Wu Y, Zdobnov EM, Zhang L, Zhu D, Gibbs RA, Honey Bee Genome Sequencing Consortium. 2014 Finding the missing honey bee genes: lessons learned from a genome upgrade. BMC Genomics 15:86. doi:10.1186/1471-2164-15-86. 24479613 PMC4028053

[81] DennisG, ShermanBT, HosackDA, YangJ, GaoW, LaneH, LempickiRA 2003 DAVID: Database for Annotation, Visualization, and Integrated Discovery. Genome Biol 4:R60–11. doi:10.1186/gb-2003-4-9-r60.12734009

[82] Huang DW, Sherman BT, Lempicki RA 2009 Systematic and integrative analysis of large gene lists using DAVID bioinformatics resources. Nat Protoc 4:44–57. doi:10.1038/nprot.2008.211. 19131956

