# Supplementary material for: Robust Transcriptional Response to Heat Shock Impacting Diverse Cellular Processes despite Lack of Heat Shock Factor in Microsporidia
Source: mSphere. 2019 May 22;4(3):e00219-19. doi: 10.1128/mSphere.00219-19 (PMC6531884; doi:10.1128/mSphere.00219-19)
Supplement: FIG S2 [file mSphere.00219-19-sf002.docx]

# Supplemental Figure 2.

#
