# Supplementary material for: Robust Transcriptional Response to Heat Shock Impacting Diverse Cellular Processes despite Lack of Heat Shock Factor in Microsporidia
Source: mSphere. 2019 May 22;4(3):e00219-19. doi: 10.1128/mSphere.00219-19 (PMC6531884; doi:10.1128/mSphere.00219-19)
Supplement: FIG S5 [file mSphere.00219-19-sf005.docx]

**Supplemental Figure 5.**

Heat shock Element (HSE) – (full site = GAANNTTCNNGAA)

**BOLD** = Transcriptional Start Site

Red = CCC motif {Peyretaillade:2009fd}

Green = TATA

**NCER_102023**

AACAAATTTGTGTAAGGATTAAATTTATATATTTCTATATTTAAGATATATATTGTGTCATCTGGGAAAAGTATTTTATTTACAATTAAGTGTTGTATAGAGAGAACATAAAATCATTTTTATTTATTTTTTCATTGCACACAACCTTAACTTAAAATTACTTTATCTCCTTTCACATTTACAATTTAAAAAGGTTTAAAAATGTTGACTTAAGATAACTATATAAATAATAATTTAAAACAAAAAAGCCAGTAAAAATCTATTTTTAAAGATACATACTGTTTATAATAGAATTTATTTTAGAAAGAAATTGAAAGCATCTATTAAATAAATTTCTTACAGAATAGGAAATAAATTTTGGTACATAAAATTACGTTAATATTTATTAAAAATAGCATTTAAATTTTTAAATATTTTATAAATTGTTTTCTTTTAAAAAAGTTTTTTCTTGAAAAAATGAATTTTCTAGAATTTTTTTTTCTATAAATAAATTTTTTTTATAACCCTGCATAA**ATGAGTGAGC**AATCTAAAACTTCAAATGCAATTGGTATTGATTTAGGTACAACATATTCATGTGTAGGTGCTTACATGAATGGTAAAGTTGAAATTATCACAAATCCAGATGGTGATAGAACAACTCCTTCTATTGTGGCTTTCGGTGAGTCTGGAAATATTATCGTCGGTTCTGCTGCAAAGAGTATGTACACAAGTGATCCTGCCTCAGTTATTTTTGATGCTAAAAGAATGATAGGAAGAGGATTTGATGATGATAATATTAAGACTTGTATTTCAAAGTGGCCTTTTACGGTTGTTAGATATAATTTCCAAACTAAAAAGGAAGAAGCTGCTCCTGTTCCTGGATCGCAAAAAGTAATTGATAATATTGCTATTAAAATCGAAAAAAATGGCAAGACAGATTATTATGCTCCTATTGAAATTTCATCAAGGGTACTTACATACATGAAAAAGGCTGCAGAAACTAGGCTTGGAACTAAAGTTGATTCTGTTGTTGTAACTGTTCCTGCTTATTTTGAAGAACCTCAGAGAGAGAGAACTAAAGAAGCTGCAATTATTGCTGGATTCAAACCTGATAATATTAGATTACTTGCTGAACCTACTGCTGCTGCTATGGCTTATGGTCATAAAAAGACTGAAGAAAATCAAAATATTGAAACTAAAGAAGATATACTTGTATTCGATCTTGGTGGTGGTACATTTGATGTATCTGTACTTGATTTTGAATTTAGTACAAAGAACGGATCTGTGGGTGTTGTAAAGGCTACTGATGGTAATACTTTCTTAGGAGGTGTAGATTTTGATAATTTATTAATTCAATATGCACTTGATGAATTTAAAAAGAAAAATGGGCTTAGTCCACAAGAAGAGTTTAAACAGAATTCAGTTCTTAGATTAAGGGCTGAGGTTACAAGAGTTAAAGCTTTGTTAAGTTCTACCGATAATTCTCAGATTTATGTTCCTTGCTTCCATGGAACTGTTGACTTAAATATTCCAATTACCCGTGCAAGGTTTGAATTTTTATGTGATCCATTATTTAAGAATTGCTTAGAAAGAGTAAAGGGATGCCTTCTTTTGTCTGAAGATATTAAAGGAGTGAATTATTCTAAAGATGGCACTAAATTATTACTTACATCTGATTTAGAAGCAAAACTTAATCAAATAAAGAGTAAGATTTCAAAGGTCATTCCAGTAGGAGGATCATCAAGAATCCCAAAGATTAAGAAAATGTTAGGAGATTACTTTGGACCAGCTAAGGTTGTTGAATCATTAAATCCTGATGAAGCTGTAGCTTACGGTGCAGCATATCAAGCTGCTTCTGTATTCTCTGATGTTATGGGTGAATCAAGTGGATTACTTCTTATTGATTGTTGTCCTTTAGATTTGAGTATTGAAACAGCAGGAGGTGTTGCAACTGTTCTTATTCCTAGAAATAGTTCTATTCCAGTTAAAAAGACCGAAACATTTACAACTTATGCAGATAATCAAACAGCTGTAACAATTAATGTTTATGAAGGTAACAGAGCTTTAGCTAAGGAAAATAATTTGATTGGATCATTTAATTTAGATGGAATTATTCCAGCACCCAGAGGAGTACCAAAGATTGAAGTCACCTTTGATGTTGATAATAATGGTGTGTTGAATATTACTGCTGAGGATAAGCAAACTAGTAAACAGTCGTCTTTAACAGTTTCTAACACTCAAAGTAGACTAACTGATGAACAAATTCAAAAAATGAAAGAAGCTGCAGCTGAACATGAAAAAGCGGATGAAGATGTTAAAAGGCTTATTACAAAGAAAAACGGATTTGAATCAATTTTACATTCCTTTAAAAGTGCAGTAGAACAACATCAACAGTTACCACAAGAAAAGAAAGATGAGATTATTGCTAGAATTAAGCCTTACGAAGATTGGGTATATGGAATTGATGGAACTAAATTTGATGAGAGTGAAATGGATCAAAAGGATCAGGAACTTAAAGACATAATGAAAGAAGTAATGCCAACCGGGCAGGCTCCACCACCAGGTGCTTAAATAAATTATTAGTTTTATTTTTTTTTAATTTTTATTTGTAAGAAATGTTTGCAATAAAACACATCAATATTTTTTTGGTGTTTAACTTTATATTCTTAGTTATACAAAAATGAAGGATTTTGGTAAGCAAAATAAGGGCGAGCAGAGAAGTGTTGTTCTATACACTTACTTAAGGTAAGAGATAGTCTTTAAAAAGGCCGACAAATTCTGTAATGAAGGGTTATGGTAAAACAAACGAGAGCAAGCAGAGAAGTCTGCAAAGTGAGTACTTTGATACTCTTTGACCTGGGGGAGGTCAGGCATGATACTGTTGCTGTATC GCCCCGCTGAACTGAGTTGGGTTCTTAAATGGTGTATTGAGCCAGGCGATGTTGTTCTGTGCACTCACTTAAGGTGAGAAATGGTCTTTAAGTAGACCGAGGATTCTGTATCCTAAGGGAACGCTCTTTTCTCCTATAAGCAAGGCCGTAAAGGTATGCAGGCGTGTAAGCGCAATATTGATGTTTTGTTC

**NCER_102035**

TTTAATTATATTAAAAAATCTTGATTAAGAATTATTAATAAGGAATTATAAATATAAACAATTTAATAAAGATTAAACTATTTATGCACGTTTAATAAAACATTTAACACTTATTTAAATCATAAAATATCTATTTACTTTATATTTAAAATATTAAATTAATTGTTTTTACACTTTTTAAAATATTTTCTTGTAATCCTTAAACTAATCTATTTTTAGTAATGAATAAAGTTGTAAGCTTCATCAATAATTTTTGTAACAAAGCATTACAATTATATAGTTAAATAAATTTACTAACAATTTTTTTAAAATTTTTACACATTTAGTGAAAAAAAGTCAATTCCAATTATTTATATGATTTTTCATGAATTTTCTAGAATTTTTTAAAATATAAAAAAATATTTTTTTTGCCCCTCAGA**ATGGAATTTT**TAGGCATCGATTTTGGATCTTATAAAACTACACTAGCATCATCAAAAGATAATGGTAAAAT TTTAGGAGACGAACAGGGCAAAAGAGCTATTCCTACACTTTTAGAACTTACTTCTCCTATAAGAAAGTTCGGTAACAGTATTACTGGTGAACATGAAGGAAGCATAAATTTAAGATACAGAAATTTTAGGGATGATTTATCAAAATCTGAAAATTGCCAAGCTTACATGATGTTTATGAAGTATCTCGATAGGATAATAAAAAATAATACTAGAGGAGCTCCTTCTATTTGTGTATCTATTCCTTCTTATTGTACGTACACAGATAGAAAATTTTTGGCTGATATTATGAAATGTTGTAATATGAAATTAGAAAGGTTTTTTAATGACATTACTGCTATAGCAATGTTTGCTTGTCTTCGTAGAGAGAAAATACCAGAAAAGTTTATGATTTTGGATTTTGGTCACGCAAAAACAGAAGCAGGTATTTTTACTTATAAAGATTTTGTATTAACACCTTTGTACATAAATAATATAAAGGTGGGTGCGCAAAATTTTGACAATGAATTAATTAATTTAATTATGCGAAAATATAGCATTCCAGATAAAATAATATGCAAAGAAAATATTTTAAGACATCTTGAAAAGTTAAAAACAGTGTTAAATTCTGCGGAAGTGGCTAATATACAGATTTATATTAATGAAACGCCAATTAACATACAAGTTACCCAAGAAGAATACATTGTAGCATGTAAAGAAAGTACTAATAAATTAACTAATTTTATTAATGATGTCATAAAAGAATCTGATACTAAATTAGTAACAGAAATTACAGGGGGTAATTCATCTTCATTTTTAGTTAAAAATATTTTAAGTGATAAATTAGATATTCAATCTACTTTAGATTTAACAGAATCATGTGCTATTGGTACAGCACTGGGACTAGCTTGTTCTGTAGTTTCTAATAAATTTAAAATGAATGATATTATTGGTAGAAGCATTTCAATAAAATTAGAAAACGCGGAAAAATCTACAGAAATATTAAAAAAAACAGATTTAATAGGAACATCTAAATGTGTTACGTACAAACAGAAGGATGCATTTAACTTAGAAATATTCGAAGATGTACTTAAGATTGGAGATTTACAAATAAATAAAGAATCCTCTGATAAAGTAGAAGCAGTAAAAATTACTATAGAAGTTAATAAACTTGGATTTATTGATGTTAAATCAGTAAAGGTAGATGATAAAGATATTGATTACAAATATACAACATTTGAAATGTCTTCGGATCAAAAAGAAGAATTGATTAATACAGAAGAAAGATATCGACTTCTTGAAACCAATATTGAAAAAATAGGACATATGAGAAATGAATTAGAAACCATGGCTATGAATTTATTAAATGCTATAACTGGAAATTTGCAGGAGCTTTTTAATGAAGAAGATGAAGAAATAGTTAAAAAGGTTGCTATGGATTTGTTTGATATTCCTTCAGTTTCTGAAATTGAAGAGGAGGTGAAAGTGCGAGATGACGTAATTAAACAGTTGGATTTTGTTAGTAAAAAATTAGAGGGATTGGAAAGTAGTATTAAGGAAGAAATTGTTAATATGAAAACATCTATAAATGAATGTAAAGATAAATTTGCCAAAAAATCGACACCTTCTTATTTTAAATTACAAGGAATTTATTATAAACTTGAAGCTTTTGATAAAAATTTACGATTAGATTTATTTACTGCTTCAAATTTTGACAAATCGCCTTTTGATGCATTAAAAAACGATTTTGATGCTGCAATTGTAAGAGCAGAGGAAGAATTGAAGAAAATGAAACAAGAAGAAGAAGAGGAAAAATTACGAGCTCAAGAACTTGAAAATAAGAAAAATGAAACATCTGAGGATGCGGAAAAAGAAGTGTCTGAAGAAAAAAATAATGAAAAGGGAAATGAAGATAAAGAAACGAATGAAGAAAAATAAATTATTATTTTAGTAAAATGATTTGTTTATATTTTTAAATTAGATCAGAAAGAGAGCTTTAAAATTTAATATCACAACGTTTTGAAATCCAAAACTCTTTTGAGTATAATTTTTTTAATTAAATTTAATTATTAAATACAAATATTTTTTTTATGAAGCTTAGGAGAATTAATTTTTTAAAAAAAAATCAACTTGTTTTTTTAGAAACCAAGCACTACAATATTTTAGTACACAACTAGTCAAGACAATCTTTTAAGACTATAAAAAACATATTTTTTAAATTTATTCTTATATTTTACTTAACATCTATATAAATAAAAAATAATTAATTAACTGCATTTGACTTGGGAGGGGTTGGGCATAATACTATTGCTTTACCACTTCGCTGAACTGAGTTGTGTTCTTAATTGATGTATTGAGCTAGGCGATGTTGTTCTGTAAACTCACTTAAGGTGAGAGATGGTCTTTAAGTGGACCAAGAATAATGTATCCTTTGGAACGCTCTTTTCTCCTATAAGCAAGGCCGTAAAGGTATGCTTGCACGCAAGCGCAACATTAATGTTTTGTTTTTACAGTGGAGTTTTTGGTGGCCTGGTGGTTTTTC

**NCER_101194**

TGGTAATATTTCTAATCGAAGCTTTAATTTTACTAAACTGAAATCATTTTGGGAACAAAAATCTATACTATAGAAGTTTATTATTGTAAAATAAAATCATTAACTATTAAGCTTTACTTTTATAAAATGCTTTATTTTAATTTTTAAAATAAAATTTTTGCTAATTTTTTTCAAGACTAACAAATTTGTTTCTAATTTAAGTCCATTATTTTTAAAAATTTTGCTAATACGCTCTGCAACTTAAAAGCTTAGGAGTAGCTTGTAGTACAAAACAATCAAATCTGTAGCCTATAACATTTCGTCTTATTATCGACTAAACGAATAATTATTTTTGCGATAAGCAAACATTTAATAATAAGTAATTTAATTAAATGACTAAGAAAAGTTATAAAGTTTTTTGTCATTACACGCTGCACATTAAACAAATGTTTTAGCAATGTTATGAAAAGCAACTTGATTCCTCAAAAAGATTATTTAGTTATGTAAAAAATTAAATAAAAAACTAGTCAGATCACAACTATTTCAATACATTAAATGTACTATCATCAGTAAGAAAAAAGAATCTTTCTAAATTTCAATAATAAAATAGTTGTCTTTATTCCTCATTCTAGTTGTACAGTTTTCATTGCTTTCACAATACTGACTCGATGAACTAAACAAACGATTCTGAATAGTAAAATCGACGTGGGGGTAAAAATAAAAATTTTATTTATAAGAAAATTGAAAAATGAGGTTGTGGGTACTTATAAAAAATATTTCTAGAAATAATTAATAATATGGATTAAATAGAATTATATAAAAATATAGTAAACAAATCTAAATTTTTTATTTTTTGGTTACTGCCAAATCATATATAAATATTAAATGTTGTAAATATTGCACCTAATCTTTTTCTACAATTAACAATTATTTTTCATTTCTTATCCCCAATGAGTGCAGTTGAAATGTCTTTTGAAAATTTAAATTTAGAAGTACCATTTATAAATCTTAATAACTTAAATAAAAGTTATGTTGACAAAGCGATTAAAACAAGAGGATTTGTGTGTAACATAAATGTTTTTAAAAAAACCACTTTTTTAACTCTTAGGGATCAAAACAGAACATTACAATGTGTTTTATCTACTACTGAAGCTGCAAAAAGTCTTAATAATGAATCATATATTGAAGTGGAAGGTAAGATTTGTTTGGTCTCGACAAAAATTAAGAGCTGTAGTTTTAGCGAATTAGAATTACAGTTACATACTTTTGCGATTTTGAATGCAAGTGAGTCTGTGTTACCATTTAGTTACAAAGACGTATCTTACACGCTAGAAGATTTAGAAAAAATATAATGTAAGTCCGGTTTCCTATCATTTGTGTTTAGATAATCGCTCTTTGTATTTACGATCACCGCAAGGTTATGCTATTACTCGAATTCTTGACGCTGTTATGTTTAAGTTTAGAGATTTTCTAAGGTCTAATGGATTTATAGAAGTTAAAACTCCTAAATTAATAGGTGGTGCAAGTGAAGGTGGTGCTAATTGTTTTAAAGTTGACTATTTTGCAAAAACTGCTACTTTAGCTCAAAGTCCACAGCTTTACAAGCAAATGTGCATTTTAGGCGGGCTGAAAAGAGTTTATGAAATAGGGCATGTTTACAGAGCTGAAGAAAGTAATATTAATAGATATTTAAGCGAGTTTATAGGTTTGGATTTGGAGATGGAAATTACTGATAATTACATCTCAGTAATTAATTTTATTTATGATTTATTTAAGTCTATATTTGTATTTTTATCCGAAAGTTATTCTTCGGAGCTAGAAACTATTAAGCAATATTTCGAGTTTGAACCATTTATGTTTACTGATAACCCTGTTATTTTGGATTATATTGATTGTATGAAGCTACTTAAGGATGAATATAACATTGATATGAAACTTGAAGATGATTTTAACAATGAAAATGAGAAGAAGCTGGGTGAAATTGTTAAGAAAAAGTGGAATACAGATATTTTTGTTATTAAGGATTATCCAGTTTGCTGCCGCCCTTTTTACACAGCAGTTGATTCTAAAACTGGATTACAAAAAGTTATGATTTTATAATTCGTGGCGAAGAAATTTTATCTGGTGCGGAGAGAATCAATTGCTATAAAACATTGAAAGAAAATATTGAAAGATGCGGTATTAATATTAGTAGTTTAGGTGGATATTTAGAAGCTTTTAAAATAGGTGCTCCACCTCATGGAGGATGTGGAATAGGATTAGAAAGACTTGTTAAGGCTTATTTTGGAATGAAAGATATTAGGTATTTCTCTCTTTTCCCTAGAGATCCAAATAGGCTTTATCCTTAATTAAAAATTTTTAATGACATTTTTGTAAAGACATACATTTAATATATTGTGGTATAAATCTTTTATAGTCTTACAGTGAACGATAAAAGAGGCCTACCTATACAAAGAAGCATACAATTAAAATTAAATGTCAGATAAAAAATTCTAGAAATCACAGGATAACAAAACTACATTTTAAAGTTTAAAATTATTATAATTATTTTTTTAATTAATTTTTATAACTGAATAGAAAGATAACATACCATAAGTAATACTAATAACTGCTAGAGCTGTGTTTAAAGAACTACCTATAACTTTTATGTTTATAAAATAATAAAGACTCGCAAATATAAAACACATAAGAGTGGAAAATGTTCCACCAATAAATTTACACATTTTATCAAAATCTATATTTGTACAAGCCAATCCAAAGCAACTACTGATAATTAAAAATGAAGTGAAAGCCCTCACATATTTGTATTCTTTATTTTTTAAAATTTTTTCATTAATCATATTTAGTAAATATTGTTTACAAGGATTTGTCTGTAAAGGAACAGAAAGTCCTAATAACAGAATGTAAAACCACGATAGTGCTGCTTTTACTGTATCATTGGGATGCGTATTAATAAATGGTTTTACTATCTGATTACCAAAAGCCATATAACTGATAATACCAAAGAGCAAGTAAATAATGCTTGCAGATATAAAACTGCATAAAGCTGTAAGTTTAAATTTCTGTGTGTTGTAAAATTTCATTTCATTTTGTAGAGTAAATATGTTTTGGTGGCAAGTAAAACTAAAAACGAAAGAACTTAAATTTTCAATATAATTATGTGATTTTCTAAAGTAAATCAAGTTTGGCAAGTTTGTAAGAGTAATATATCTGTAATATGATGTACTTATTAAAAGAAGTATCGCTATTAATCCAAAACTGCTAGTATATTTCAACTTATCGAGCTTAGTCATAAAAATAAAAGGTGCAATAAAAAGTATTACAACAAAAAATACAAAATATAAATTTACGTTTAGATTATAATACTGAATTAAAAAATTAATTTGACACTTTATGTACACTAAATAAGCTATAGATACAGTATAACATTTAAAAATCACTACAATGTCAGCAAGATATTTTAAACATGGCATAAAATGGCGTGTGACGGTTGATAACGTATTATTGCGTCCAAATCTTGCATTAAGTTCAATATATAATAGTATTCCTGCATAAGAAGAAAATGCAGATATAAAAGTCAAAAATATGCTAGAAATTATTCCAAATTCAAGTACTAAAAATGGATAATTTAGTATTCCACATCCCATAGTTGTTTTTAATAAGTTTACGTAAATGGATAAAATTTCATGATTTGACTGTTTCATAAGGGGCAAATGTATAAATTTTGTACAGAATAAAAGGAGACAAGAAAAACTTTATTTATTTTATAACTACTTTGACATTGCATAAGTATTAATTTTAAAAAACGATTATTTTTGTTTTAAATAATTAATATATTTAATTAAAAGTTAAGTCGAACTGCAGATCAATCTTAAAATTAATATTTTGCGCATTATTATGTAACTTATTTTACATAAGAAGGTAAAAATTATCAAAAAATTATAATTTTATTTATGTGTATAAAAATATCTTATTATTTTTATGAACGTATAACCAAGGCATTCTACAATCAAAATAAATATTTTTACAAGTACCTTGGATCCTATTCACAAATGAATATTGATTTTCTCCAATATTAACATAAAAAATTAAAATTTCATTTTCTGTGGAAACTGCTAAAAGAGAATGTACATCATTATAAGATATTTTGTAAAGATTATTATCTTGTACCATTGTATGAATTACAACATTTCTTTCATAATCAAAAATAAATATTTTTTTTAGATTATTAATTAAAAAAATTAAATTTTTATGAACAACAAAATCAATAATAAACGAAAAAATCGTAGATTCATTAATTAAAAGTTTCCTAGTTATGTCATATACTTTTAGACTATTCGATGTAATAACAAATAATAAATCATTATTAGTAAAAATTTTTGTTAATTTATCTAGTTTCTTAATCGGTACTTGAACTTGTGTATTAAAGAAATTAATAGATCTAGACGTAACATACAAATTGTACTTATCACAAGAAAAATCAAATACATTAGAACTTTCATGTAAAATTTTTACTGACTTATCTCTAATATCAACCTCATAAATCTTAGAAATTCCATTCTCTAATGTACATAAAACTCTGATAGAAGACTCAAGCTCAACTTTCTCAATTATTCCATCAAGTTCATGTGTAAATATCCGATAAAAATTTTTTATTTCAAATATTTCTAACAAATTATTCGTGTAACAAGCAAAATATTTCCCATTTCGATCAATAACACACTTATCTTGTATTTTATAGTATTTAGCAAAAGTATGAGGAAAGTATAAATCTTCTTTAACAATGTTATTTTTTAACAAATCGCTAATTTTTAGTGTTTTTCTCTCTCTAGGAAGATATTGTTTCAAATAAGTCCTTCTTAATTCTTTTTCTAAATCCTCTTTTTTCATTCTTAAATCTACTAATTTACCAGTATATTGATCATTAACGTCTAGTACAATATTTTGATGATAGTCTTTAAGATCATCAACCCAAATATCACTTATTACGTTTTTGGGTTCTGGCGCAATTTTAGTGTAATCGTTCTTTCTTAAAAACTTTTTAACCATTTTTTCTTCGTTTAAAACACTGGATCCCTTTTTTACAAATTCTAAAGGTGTATTCTGTGTATCTTTAGAAAATGTTAGTTTGTAAGGGATATACATGTCTACTGTAGGATCAAGACAAGCATTCTTTTTGTATTTTCTTACAACTTTAATATCTTCTTTAGTTATTTCATAATATTTATTGTTAGTTTTAACAACTCTCTTTGTTTTTTGTTTTCTCCTGTTCAAAAACTTATCTATTCGATCCATTTAACGGTTAAAAAATTGATTGAAAAGTAAACATTTTTGCATTTCAAGATTAATCTAGACCCCTCATGACAAAAATAAAAATAAAAGGGATTTCTGTAGATTTTCCATATGAACCCTATGAGTCACAGAAACAAACAATGGAAAAAATTTTAACTTGTATGATGGAGGGATCTACTGGAATGATAGAAAGTCCTACAGGAACCGGTAAGAGTCTTAGCATTTTATGCGCAGTACTTGCATACAAGGAACATATAAAAAGGAATAACATACCGCTTCACAAGCCACCTAAAAAACCTCTTTTAGATGCCGTTGATGAATCTAAACAATCAGATATTAACGAGCTTCTTAATGCTACACAAGAAAAGGCTGAAAATGATAATGATTTCAAAATTTTTATATGTTCTAGAACACACAAGCAATTAGATCAGCTTGTACAACAACTTAATAAAACCAGATATAAACCAAGAATATCAATTTTAGCCAGTCGTAATCAATATTGCATTCATCCTAAACTACAAAATGTTACTGATAAAGCATCTGCATGTTCTGAATATATTAAAAAAAATCAGTGTAATTATGTTAACGGCAAAGATAGATTAGCAAAAAGAGTTGGACAAAATATTTTTGATATTGAAGAAATTGTACGAGAGGGAAAAAGATGCGGTGGATGCCCATATTTTGCCGCACGAAAATTGGCAGATGATGCTGATATCATTTTTGCGCCTTATAATTATCTTTTAGATCGAAATGTCAGAGGAAATACTGCGATTGAACTGTCCAATTCTATTATAATTATTGATGAAGCTCACAATATTGATGATGTTTGTAGATCATCTGGATCAATAGAACTTACATCTAATATTATTGATATTATTGTCAACGAACTTTTAAATGCTGTAAAAAAGTCAGCATATCTTGGTGAAACAAAAGGGGATTATCTAATACTTTTAGAATTATTTAGAAAGTTAGTATTCAATGTTGAAAAAGTTACATCTTTTGACAAGACAAATAAAAACGTTAAATTAAGAATACGAAAAGGAAAAAACATTAAAAACGAACTTATAGAAATGACTATTACGTGTGAATTTATGACACAATTTAAAAATGCTATTTATGGTATAGAGCAGGTTCAAGATGGCAAGTCTCTTGTAAGTGTTAGTACATGGCATATAATTGAAACATTAGATTCAATCCTTAGTCCTCTTCTTTTTTCTGATTGTGATGTGTATTCTTATGCGTTTAATAAATGTGAAAATGATTTCCAAGGACGAACTTTTTTTTCATATAATTTTTGGTTAATGGATGGTGCGTACGTTTTTTCGCCATTTGTAAAGCAAGTTAAAGCTCTCATTCTACTTTCTGGTACATTAACTCCTTTTGTGTCATTTTCTAGTGAACTTGGTCATAAATTTGCACATCAGATATCTGCTCCACATTTAATTAATGATAAGCAAGTACTTGTTACATGTCTTAAAAGAGGACATTTAAAACAAGAACTGATTGGAACTTATAAAATAGCTGAAAGCTTGGCGTATTTAGATCAAATTGCTAGAGTTGTGTACGATACGGCCTATAAAGTACATGGACATGGAGGAACGTTAGTTTTTGTGCCATCTTATACATTTCTTGATAATTTACATGCGCGAATTAAACTTTTAAGGCTTGATAATTTATTTTGCGAGCCAAAAGCAGGTGGTATAAACGAGTTTGAAAGCATTTTGAAAAAGTATCACAATCGAATAAATGAAAAAAAGCCAGTAGTACTGTTATGTGTGTATAGAGGCAAAGCATCAGAAGGTATTGATTTTAAAGATTCGTCAGCCAGAGCAGTTATTTGTGTAGGAATACCTTATCCTAGCCTTGTAGATCCACAAATAGAATTAAAAAAAGAATTTAATGATAAACACAAACATTTTAATGGTCGTAGATGGTATGAAACACAAGCTTTGAGGGCAGTTAATCAAGCTGTTGGGCGAGCCATTCGGCACAAGGACGACTGGGGCATAATTATCATGCTTGATAGTAGATACAGCGATAAACGAGTATCTGTTCAATTATCAGGATGGGTATCGCAATTTTTAAAAGTACATAATGACTATGAATCGTGTATAAAAAGCATTAATTTATTTCTTTCGGATAAAAGTAAACAATAAATATTTTTTTTACTTTTAAAAGAGTATTTAATATAAAATTTTGTCAGGTATTTATTTATATCGAAACTGTTATAATTAACTTTTCAAAAGCATATAAATATTCTTGGCAATGAGTAAATTTTAATATTAGTTTTATATTTATTTTGTCATATTACAAATTAAAATTTTAATAAATTATCTTAAAACACCAACACATTTCAATTAATTGTATTAAATCTTTGATAAAGCAGTTTAAAAAACAAACCTGGTATTTTAATCTTCTTTACTTTTTGTAAAAACTTAAAATACTAATTATTAAAGTTTTAAAAGTAAATGATTTTAAAGCCATAAAAATTTCAATTAAGTTATTTGTTAATGAGCTCTTTCATTTAACCATTTATAATGTAACAAAACTGCTATTAATAAGAATGTTTATAAAATATTTTTCTATTTTGGTGTCACTATAAAAACAAAATATTACTAATTTTTTATTCAAAATTATTTTCTAGAAAAAATGAATTTTCTAGAATTTTTTTTTCTATAAATAATTTTTCACCCATAATCCTTCA**ATGTCAAACG**AACAAAATAAAGAACAATATGAATTTGATGTAAATACAAATCAACTTATGGATATCATTATCAAGTCGGTTTATTCGTCTTCAGAGATATTTCTTAGGGAGTTAATTAGTAACTCGAGTGATGCATGTGATAAATTTAGATCATTGTATTTAGAGTTTCAAGAGCAAGGAGTAGTGACTGATAATCCGTCAGTTTTAGAGATTCAGGTGATTCCAGATTTAGTAAATAAAACATTAACTATTAAAGATAATGGTATTGGTATGAAAAAATCAGATCTTATAAGTTTTATTGGTACTATTGCTAGTTCTGGTACTAGAAAATTTAAAGAAGCTTTAGAAAAGAAAGAAGGTGGAAGTGGAGATATTAATCACTTAATTGGACAATTTGGTCTTGGTTTTTATTCTTCCTATCTTGTTGCAGAGAAGGTAGATATTATAACTAAACATCCTCTCGATGAAGCATTTGTATGGTCATCGAATGGAAGAGAAAGCTATACTATTCAAAAATATGAAGGTGAAGACTTTAATCATGGTACATCTGTAATTTTATATATTAAAGAAGGAAATGAAGAATTTTTAGAAAGTAAAAAAATTATTTCTTTGATAAAGAAACATTCACAATTTGTTCTTTATCCGATATTTACATACGAAGAAAAAGAAGTAGAAGTTAAAGATGAAGAAAAGAAAGAAGTTAAAGATGGAGAAGAAGGCAATGTTGAAGAAAAGAAAGAAGATACTGAAGAACCTGTGGTTGAAGAAGTAGAAGAAAAACCAGTTAAGAAAACAAAAGTTACTGAAAAAGTTAAAGTGAACACTGATAAACCGCTTTGGGAGAAAAATATCAAAAATGTACCAGAAGAAGAAATTACAAGTTTCTATAAATCTATTTCTGGAGACTGGGATAATTATCTTGCTGTTGAATCTTGGACTATTGAAGGTTTTATGACTCTTAAACTATTGCTTTTTATTCCTAAGAGAAGTAGAATGGATATGTTTTCAGGTAAAAATAAAAAGTCTAATAATATAAAATTATATGCAAATAATGTATTTGTTACTGATGATTTTGGTGATAATATTCCTGAGTGGATGAACTTTGTTGTTGGTGTTGTAGCTAGTAATGACATTTCGATGAATGTGTCAAGAGAACTAATCCAAGGACATAATGTTATGAAATTGGTGAAGAAAACACTTCCTCAGAAACTTATGGATATGATTACTAAACTATCAAGAGATGAAGAAAAATATTCTGCATTTTATAAGGAATTTGGTAATTGCTTGAAATTGGCTGTTTCAGAATGCACTGATTCTCAACAGGAAAAACTTGTCAACTGTCTTAGATATGAAACTTCTAAGTCTGAAGGTAAAATGAGATCATTTAGTGAATATGTTGACAATATGAAAGAAAATCAAAACCAAATTTACGTTTTGACAGGATTATCCAAAACTCAAGTAGAGCAGAATCCAATGTTAGAAGTATTTAAAGATTATGAAGTAATTTTTATGTATGATTCTATGGATGAAGTTATGCTTAGAGGACTTAAAAAATATAAAGAGAAAGCTGTTCAGAGAATTACTTCAGAGGGGGTCGAAATACCTGAAGAGGCTCAAGCGAATAAAGAAGAGTTAGAAAAAGAGTTTGAACCAGTAATAAATAAAGTTAAGGAAATATTAGGAAGTAATGTAGAAAAGGTCATATTGAATTTTAGATTGGGTTCATTACCTTCTGTTATTTCTACAACAAAATATTCTAACTCTGCTGCTATGGAGTCAATCATGAAGTCTCAAATTATTGCTGAATCTAATCCACTTGCCGCTATGGCTGCTACATCAAAAAAGATTTTTGAGTTGAATCCATTTAATCCAATTGTTGTAAGAATGAAAGATTTAGTTGCTGATGAACATAAAGAAGACTTTAAAGAAATCACCAATTTGCTATTTAATACTATGCTTTTAAATTGTGGATTTGTTTTACCTGATCCTCAGAGCTATTGCAGTACAATTTATAAGTTTTTATCTAAAAACAATCATTAAATGCGTTTAAGTTATTTTTTAATTTTTTAAAAGGTCTCACAATAATAAGCATGAATAACAACTGCAATGATTCAATAAAAAGGCGAATTACAGGCTTGAATTAAAAAAGGTAAAACGGGTTAAATATGCTCTCCGTTACAAAATTAAATTAAGACTTATTTTATTATACACAAATTTTACTTATAAGTTTAAATCTAGAAAAATAGCTACAACGATATGCTGTCATAGAAATTGTAATGGTTCTGCTTAACAGTAGGCAAATGGATTAAGTACTTGATCATCAACGTCTACATACCACAAGACAGGGACCTCAAGAGAGAAGTTCTTGCCAGACTATTATCCCTTCTTTATGAAGAGAAATTAAAAAATCACTATAGTGAGATCAAGAAAAACCACATGGTGTTCTAGAACACAAAGGTGGCTAAAAAGATTTGTCGGTAACGACTATGAAAATGGTGATAGCAGACTTATCACAACAAAAATAATTGATGTACTACGAAGTAGAAGAACTCTGAACATTTCAGTAGCTGCATCGATAGCAAATAAGTATGACACAAAAGATTCACAAATCAAATATGTCATACAAAAAGTTAACAATCGAAAGATTAGAACGTATACTAGATTAAGATG

**NCER_100860**

ATATATTTTACAAAACAATTGAAGATGACGGAAGTCTTAAATCTGTAAAGGCTGATCCATTTCTACCAGTAGAATTTTTTTTAGTTAAATTAACACATGGTAACAAACAGAATCCCCTTTTTCTCAATAACACACCCTTCTATGGTTCAGTTACTAATAAAAAACTTGCAGAATATTTTAATGAAGATTATTCATTTGAAAAATTTTCTAATTTTGATTTACTCCAGAGACTAAAAGATAAATTTAAAGACTTCAATGAGTTATTAAAATGCATTGTTGAAAAAGATAGAAATTATTTTAAGTTTTTTACAGAACAAGAAAATTTTAAAGAGTTTACTAATTCCTTACAAAAATATTATAAAAGATCGTGGATATGTGCAGCTTGTACGTTTATTAATGAGAAAAACTTAGATCGATGTGAAATATGTGAAACACCAAACCAATAAATTTTATAAATTTACTAGTTTTTTAAATTTATTTTTTTCGGAATTTTCTAGAATTTTTTTTTCTATAAATAGGTGGCCCCTAAAAAATGGCATA**ATGGCACAAGACCCCCAA**GGACTTTATGAAGTATTAGGACTTAAAGCTGGTGCAAGCATTGATGAAGTTAAAAAAGCATTTACAAAAAAACAAAGGGAATGTCATCCCGATGGTGCATTTTTTAAAGCTGCTCTTAGAAAATGTAAAACAGATGAAGAAAGAGCAAAAGTTGAAAAAGAATTTAAAGAAAAATCACAAAAATGTAATCAAGCTAAAGCAGTTTTATTTGATGAGAAAAAAAAGCAAGAGTATGATATGGGAATGACTGGCGATTTTGGATTTTCTGCAGGTGGTGGAGACTTTTTCGATATTTTTTCAAGTTTTACTGGTGGAGGAAGAAGAAATCGAGTACAAAAAGTTCAAGATACAGAATACGAGTTTAATATTTCGTTGAAGGAAGCTTATATGGGAAAGCGAGCTAGTTTTAATGTAAAAGTGCAAAGAGAATGTTCTGCATGTAATGGTAAAGGAGGAGACAGTGTTGAAACATGTGAACCATGTAAAGGTAAAGGTAAGGTAGAACATCATAGGAGACTAGGTCCTTTAATTTCTGTACAAGAAGCACCTTGTAATAATTGTAATTCTACTGGGTTTGTTGTTAAAGGAAAGCTTTGTCAAAGTTGTAAAGGTAAACAATACGTTGAGACTAAAGAACATATTGAAGTGGAAGTAGAGCCAGGAGTACAGAACGGTAGAAAATATACATTTTCTGGCAAAGGAAATCATAAAAAAAATTGTGTACCGGGTGATGTGATTCTTATTGTAAACATTACAAATGATCCAAGATTTAGAAAAAGTGGATATAATTTAATTAGTAAGGTTGATATTCCATTGTATGTTGCTTTGGCAGGCGGACAGATTATATTTGAACATATCACTGGTAAGAAATTTGAAATTACTTTATATCCATTTAAAGATTTAAAGAAATGTATTATTTTAAAAGGCGAAGGTTTTAAAATAAAAAATAGTTATTCGGGTGGTGATCTTATCCTCGAACCTAATATAATTATAGATAAGAATATTGATAAAGATTTATTAGCACGCGCCTTGAATTATGTTCCTGTAAGAAAAGATTTTACAGGAACTGCTGTTCCAAAATATGCAGAATTTGGTGTTATGCCTAAAGAACAGGAAGAACAAAACGAAAGAGAAAGATTCGAAGGTGCAGAAGAGTTTTTTGGAGGAAATGGTAGAGATTTTTTTAGCAAGTTTGGATTTTTCTAATTATAAAATAAATTAAAGGTTTAATTTTTTTAAATCTATGCATATTGACATATTTTTTAATTTTTTTTCTTTGTAGTTGTTTAATAATTTATACTTATTTGCAAATAAATTATAAATTATTTTTTATGTCCCTTAAAATGATAGAACGGGAATATTATGATAACGAAGTTTTCGAAGAACATAATGTATGGAGAAAAAATGTACCATACATGTATGATTTAATGTTCTCTTACGCCTTAAAGTGGCCTTCTTTGTCAGTACAATATTTTCCTGATAGTAGAAGAGATGATAGAAAAGAAAGCACTTCTCAAAGATTGTTACTTAGCACCAACACCAATGGTGAAGAACAAGAATATATTCATATTGCATCTGTCGAGTTTCCTGATAAATATGATGAATTGTTAAGTGATGATTGTAATGGAGATCTAAGATTTAAATTTGAACAGTCTATACCAGTGCACAGTTCAATTAATGTTGTTAGGTACAATCCTGTAGCATTTCATTTACTTGCTGCTAGATTTGACACAGAAGACATTCA

**NCER_101322**

CTCTTCAGGATCATCTTATGCACTCTGGCTTGTTCATGGTTTTATTTCAATACCATTACTAAACTACTTGTGAAATTGTTTTACACATCATTCTATGCAAAATAATTTCGCTCCTATATTTGTAAACGGGAACTTATGACTTTTAAACAAATCGCATTTGTATTATTCTTCACTATGATTGTTTTAATAATTAAATTGTTATTTTTTATTTAACTATCAAAATATTTCGTATAGTACAATAAAAGACGTTATAATGTTTTATTTTACGTTGTGACTTTTTAAGAATATTATAAAAATGTTTATTTATCTTTTGTAAAATAACAATCCATAGAATATATAAGTCATATCTACAACATACAAGATATATTTTTCAGAAGGATAACAACCAACCATATTTGAAAATGAAAGAGGAATTAATCCATAAATAGCAAACTGTGAATTAATAAACAGTGCACTAAAAAGGGAACCTATTGTTAAAAAAATATAAAAAATAAAAGCAGAAGAATCAACATAGCTAAAAAAGTCATCTTCACTAAATATATATTTCTTACTTAATTTACTTTCTGATTTTTTGTTCATGCTCTTTAGGGTATTATTTACACTAAAAATGCAAATAAAGAATAAATCATTGGCACTTAAAAAATCAATAAGTTATAAGCATGTGTAAATTTTTATTAATAATTAAGTAATTTAAATATGGAATAATATAATTTTTGTAATGATACTGTAATCCGTTTATTTTCATATTTTAGTTTGATAATCCAATTAATTTAAGTAAATTTATTTGTGCTTAAAATAATATTTAATTTGAGTTATAGACGATAATAAAATTTCAATCATTTTATTTTTTTTAAGATGATTGTAAGGTAATAATATCTTTGTTATATTTTAGTGTCAATACATTGAAATTCAACAAGTAATAAAAATATCATTTAAAATAATTAACATTATTTATACAAAGTTAAAAACTTTTTGAAAGCATAAATGTTCAAACAAAATATATAATTTTTTTTTAAAAATTAAAATTTCTGTGCATAAACAATACAGTGAGGGACGATTTATTCCTCCCACTAAAATGTAGATTTTATCCTTCTAATAAGGTTAAAGACATCTCTCGTCTAAATTAAAAGCAGTTTAAGAGATATATCGGGGTTCGTTGCTAATTAAAACCTATAATCGAAGTATTTAATTAACTTGTGTTTTTTAAATAATTTTTTTATTTGAAAAGTTATTCATCCTTGACGATTTTATCCACCCCCTTTTGAGAAAATAGTAAATAAAATTGCCTTCCGATGTATAACTTAAAAAATAATTTTAAGCAAAGCCTGGCACAAAAAGATTAAAAAAGCATTACAACTTAAATTATACATTTTGCTGTCGTAATAATTTTTTATTTTATTGTACATATTTACTAGATGTTATTCACAAAATTGTTCTATAGTACAAACGCAATAAATTTTTTAATAACACAGAAATTTTACAGCTAGAAGTTATGTAAAAAATACAATACTTTTGTCAGCCAATCTGCATAATGTTGTAAAGTTCTTTTTAGGAATATTTGCTAATTTTTTCATAAATTAAATAAAATTAATTAAAAACGCAATTTGATTAATTTGACACCTTGGCAATAATGTTTTAACGCTTTTATTAAAACATGCCAATAAATTATTGGCAAATCATTTGGATCAATAAAATCAAAGGTTTTAACTTAAACAATAAAAATATTATTAAATCCATTTCCTACAAAAAGTCTTCAGAATGTAACATTAAAGTTTTTCACCAATTTGTCTATTTTTTCAGGAAAATTCTAGAATTTTCTAGAATAAAAAAAGTATATAATAAAAAAAAATTACACCCCT**ATGTCACAAAATAAA**TGTACTAATAAAGCACGTGAACTTATTGAACAGGCAATGGCAAAGGCAACTCTTAATAGAAACACACAATTAGAACCAGAACATTTTTTAAACGTACTACTTGAAGATTCAAATTCTATTTTAAGAAAAGTTCTCCCCAAAGAAGAAACGAATATTTGGATTGATAAAATTATCAATAAAATTAATACATTTGGTAAGGCAGGTCAACCAGTAGAACCACAATTTACTTACAAAATATCACAAATTTTAAAAACTGATGATGAATATATTTCTGTTGATTCTATTTTAATTAATATTCTTGCATTAGACAGCATAAAGAGCTATTTAAAAGATGCAGAAGAAATAATAAAGAAATTGAGAAGTTTTAGAGGCAATAAAAAGATGGATAATGTAGATGCGGATGATACAGAAAATGTAATGTCAAAATTTGCTGTAGACATGGTTGCACAAGCACGCCAAAATGTTTTTGATCCTGTTATTGGACGTGAACAAGAGATTAGAGAAATTATTGAAATTTTATGTAAAAAAACAAAAAGTAATGCAATTATGGTAGGAAAACCTGGTGTGGGTAAAACTGCTATAGTAAATGGAATAGCACAGAGGATAGCAAATGGAGATGCACCTGGATTAAAAAATGCTAAAATTTATAATGTAGATATTGGAGGGATGGTAGCAGGTGCCTGTCATAGAGGAGATTTTGAACAAAGATTAAAGGATTTAATTAAAGAAGCCGAAACAACCCCTGGTGTAATTTTATTTATAGATGAAATACATATTATTTTAGGAGCAGGTAAAACATCTGATAGTGCTATGGATGCGGCTAACATGCTTAAGCCAGGTTTAGCAAATGGTTCTATAAAATGTATTGGTGCTACGACAGAAGATGAATATAGAAAATATGTAGAATCAGATCCTGCATTTGAAAGAAGATTTGTACAAGTTCCAATAAGAGAACCTTCAGTTGAAGATTCTATTACAATGTTAAGAGGTATAAGAGAGAGAATGGAATTACATCACGGTGTAAAAATTAGTGATAATGCTTTAGTTTATGCAGCCAATGCTTCTAAACAGTATATTCCTAATAGAAGATTACCAGATATAGCAATTGATTTAATTGATAGTGCTTGCGCTAGTGCTGTTATTTCTTTAGAAAGTCAACCGAAGGAAATATTAGAAGCAAAAAATAAATTATGGTCTTTAGAATTAGAAAAGACAAGTTTAGAAATGGATTTAAAAAATACGCCTAATTCTGAAATATTACTAAAGAAGTTGGAAGAGATTCAAAAAAGGATTGAAGTTATTAAAGAATCAATGATACCTTTAGAAGAAAATTATCAAAATGAAAAGAAAGACATTATACAAGCAAAAGAATTACGTAAAAAATTGGAAGATACAAGAATTAAATTACAACAAGCAGAAAGAGATAGACAGTCATACTTGGCTTATGATCTTAAAACAAATGTTATACCAATTCTAGAAGAGGAATTAACAAAATTAACAGGAGTAGAAATAATTGAGACACATCATGTTGCAGAAAAAATAAGTAATTGGACAGGTATTCCTGTTAAGAGATTGACAATGAAAGAAAATGAAAGATTATTGGGAATGTCTAATAGGATTAAACAGCGAATTTTCGGGCAAGATGAAGCAGTTGATACTATAACTGCTTCTATATTGCAATCGCGTGTAGGTCTTGCACGAAAAGATAAGCCTATTGGTGCTTTTCTTCTTTTAGGACCAAGTGGTGTTGGAAAAACAGAATTAGCAAAAGCCGTGGCTGCTGAGTTATTCGATGATGAAAAGAATATGGTAGTTTTAGACATGAGTGATTATGGAAATGAATTGTCTGTTACGAAATTAATTGGTGCATCTGCGGGATATGTCGGTTATAACGAAGGAGGATTTCTTACAGAACCAATTAGACGTAAACCTTACAATGTAATTCTTCTAGATGAAGTTGACTTGGCTCATCAATCTGTACTTAATGTTTTATATCAATTACTCGATGAAGGTCGTATCACCGATGGAAAGGGTGTTGTAGTTGATTTTAGAAATTGTGTAATTATTATGACTTCTAATCTTGGACAACATGTAATTATGAATTCTAATGGAATTGGAGATAATGAGAAAGTAGAGTTAGAACAAATGGTACTTAAAAGATTTGGTCCACCATTTGTAAACAGGATTGATAATGTGATATATTTTAATCAGCTAGATTATGCATGCCTTGGTAGAATTTTAGATTATCAAATTAATGAATTGAATAAAACACTAGAAGAAAAAAATATTAAATTTGCTATTAGTAATGCTGTAGCTCAAGAAATGGTTTTAAAAGCGCATTCTTCTGTTTATGGTGCGAGATTAATGAAAAGGCTTGTACAAACGCATTTTACAAGTGCTCTGACACAATTTTTATTGAAGAGAACTGATAATGAAAATTTGTATATTAAGTGTTTTGAAAAGAATGAGAATCAGCCAGGTGAGTTAGTGGGGGATTATGTTTACCAATTTCAAAAATTTAATTAAATTTTATATTTTATTTTAATTATATGTTTTT
